# Extent, characteristics and policy applications of Key Biodiversity Areas

**DOI:** 10.1002/brv.70144

**Published:** 2026-02-19

**Authors:** Stuart H. M. Butchart, Olivia Crowe, Tom Scott, Andrew J. Plumptre, Megan Eldred, Ellen McKee, Zoltan Waliczky, Ibrahim Al Hasani, Adrián B. Azpiroz, Daniele Baisero, Fred Barasa, Alison E. Beresford, Bezeng S. Bezeng, Charlotte Boyd, Thomas M. Brooks, Graeme Buchanan, Gill Bunting, Sofia Capellan, Neil Cox, Tim R. B. Davenport, Tammy E. Davies, Gina Della Togna, Wendy Elliott, Matt Foster, Jo Gilbert, Jonathan M. Handley, Diego Juffe‐Bignoli, Victoria R. Jones, Naomi Kingston, Jeannot Kivono, John Lamoreux, Penny F. Langhammer, James Lewis, Bruce Liggitt, Daniel Marnewick, Paul Matiku, Erin McCreless, Rachel A. Neugarten, Catherine Numa, Mark O'Brien, Madhu Rao, Ciara Raudsepp‐Hearne, Justina C. Ray, Samridhi Rijal, Jon Paul Rodríguez, Jurgis Sapijanskas, Wes Sechrest, Martin Sneary, Andrew Snyder, Konstantina Spiliopoulou, Thomas Starnes, Cecilia Tobar, Gautam Surya, Marcelo F. Tognelli, Andrew W. Tordoff, Maria G. Toscano, Yongyut Trisurat, Amy Upgren

**Affiliations:** ^1^ BirdLife International David Attenborough Building, Pembroke Street Cambridge CB2 3QZ UK; ^2^ Department of Zoology University of Cambridge Downing Steet Cambridge CB2 3EJ UK; ^3^ KBA Secretariat BirdLife International David Attenborough Building, Pembroke Street Cambridge CB2 3QZ UK; ^4^ BirdLife Middle East Regional Office Building 4, Bakr Al‐Baw Street, Dahiat Al‐Rasheed, P.O.Box 2295 Amman 11953 Jordan; ^5^ Instituto de Investigaciones Biológicas Clemente Estable Montevideo Uruguay; ^6^ BirdLife Africa Regional Office Westcom Point Building, P.O. Box 3502 Nairobi 00100 Kenya; ^7^ Royal Society for the Protection of Birds The Lodge Sandy SG19 2DL UK; ^8^ Department of Life and Consumer Sciences, College of Agriculture and Life Sciences University of South Africa Private Bag X6 Florida 1710 South Africa; ^9^ BirdLife South Africa 257 Main Road, Claremont Cape Town 7708 South Africa; ^10^ International Union for Conservation of Nature 28 rue Mauverney Gland 1196 Switzerland; ^11^ World Agroforestry Center (ICRAF) University of the Philippines Los Baños Los Baños Philippines; ^12^ Institute for Marine and Antarctic Studies University of Tasmania Hobart Australia; ^13^ BirdLife Europe and Central Asia Regional Office Stichting BirdLife Europe c/o Hive5, Cours Saint‐Michel 30 Brussels 1040 Belgium; ^14^ IUCN‐Conservation International Biodiversity Assessment Unit IUCN North America Regional Office 1630 Connecticut Ave., NW Suite 300 Washington 20009 DC USA; ^15^ Re:wild, Africa Program Box 2125 Arusha Tanzania; ^16^ The Amphibian Survival Alliance Panama Office Panama Panama; ^17^ WWF International 118 Dagoretti Road, Karen Nairobi Kenya; ^18^ Re:wild PO Box 129 Austin 78767 TX USA; ^19^ Durrell Institute of Conservation and Ecology University of Kent Marlowe Building Canterbury CT2 7NR UK; ^20^ Conservation International 2011 Crystal Drive, Suite 600 Arlington 22202 VA USA; ^21^ National Fish and Wildlife Foundation 1625 Eye Street NW, Suite 300 Washington 20006 DC USA; ^22^ Rainforest Trust P. O. Box 841 Warrenton 20188 VA USA; ^23^ IUCN Eastern and Southern Regional Office Pretoria South Africa; ^24^ Nature Kenya P.O Box 44486, GPO Nairobi 00100 Kenya; ^25^ Wildlife Conservation Society 2300 Southern Blvd Bronx 10460 NY USA; ^26^ IUCN Centre for Mediterranean Cooperation Marie Curie 22, Campanillas Malaga 29590 Spain; ^27^ BirdLife Pacific Regional Office 10 MacGregor Road, Suva Fiji, GPO Box 18332 Suva Fiji; ^28^ IUCN World Commission on Protected Areas 28 rue Mauverney Gland 1196 Switzerland; ^29^ Wildlife Conservation Society Canada 344 Bloor Street West Toronto ON Canada; ^30^ IUCN Species Survival Commission 28 rue Mauverney Gland 1196 Switzerland; ^31^ Provita, Calle La Joya, Edificio Unidad Técnica del Este Chacao Caracas 1060 Venezuela; ^32^ Centro de Ecología, Instituto Venezolano de Investigaciones Científicas km 11 Carretera Panamericana Miranda 1204 Venezuela; ^33^ Global Environment Facility P N8‐800, 1818 H Street NW Washington 20433 DC USA; ^34^ Department of Ecology and Taxonomy, Faculty of Biology National and Kapodistrian University of Athens Athens GR‐15784 Greece; ^35^ IUCN The David Attenborough Building, Pembroke Street Cambridge CB2 3QZ UK; ^36^ BirdLife Americas Regional Office Av. República E7‐61, Ed. Titanium Plaza, Of. 8‐2 Quito 170518 Ecuador; ^37^ American Bird Conservancy PO Box 249 The Plains 20198 VA USA; ^38^ Critical Ecosystem Partnership Fund 2011 Crystal Drive – Suite 600 Arlington 22202 VA USA; ^39^ Faculty of Forestry Kasetsart University Bangkok Thailand

**Keywords:** conservation, Convention on Biological Diversity, IUCN Red List, protected areas, Kunming‐Montreal Global Biodiversity Framework, Sustainable Development Goals

## Abstract

A global standard for the identification of Key Biodiversity Areas (KBAs) was published 10 years ago to provide a unified set of criteria for identifying ‘sites of significance for the global persistence of biodiversity’. We review the initiative's origins, the KBA identification process, characteristics of the current network, threats, policy uptake, private sector applications and future priorities. KBAs are identified using criteria with quantitative thresholds relating to threatened or geographically restricted species or ecosystems, ecological integrity, biological processes, or irreplaceability. These criteria can be applied in terrestrial, inland water, marine and subterranean environments, and to all taxonomic groups. A total of 16,596 KBAs covering 22.1 million km^2^ has been identified, with 29% of these sites in marine and 26% in freshwater ecosystems. KBAs range from 0.001 km^2^ to 712,457 km^2^ in extent, with a median size of 141 km^2^ and a mean of 1,364 km^2^. Most (63%) qualify due to the globally threatened species they support, with 48% being important for biological processes and 39% for geographically restricted species. KBAs have been identified for 18,365 qualifying species in total, of which 37% are plants and 32% are birds. The most prevalent threats are biological resource use (hunting, logging, fishing, etc., impacting 40.8% of sites with available data), unsustainable agriculture (40.7%), human intrusions and disturbance (38.4%) and natural systems modifications (water management and fire; 33.4%). KBAs are important for delivering ecosystem services to people, both locally and globally. KBAs have had widespread impact in informing protected area designation in all regions. In total, 10,054 sites (62%) are covered completely or partially by protected areas. Hence, KBAs are highly relevant to Target 3 (and other targets) in the Kunming–Montreal Global Biodiversity Framework, and to Sustainable Development Goals 14.5, 15.1, and 15.4. Indicators based on KBA data are therefore being used by the Convention on Biological Diversity and United Nations to track progress towards these targets. Many companies and financial institutions use KBAs to assess their exposure to nature‐related risks and to identify opportunities for site‐level, nature‐positive actions. Future priorities include expanding and updating KBA assessments, and strengthening efforts to protect, conserve and safeguard these sites effectively.

## I. INTRODUCTION

Site‐based conservation is a key component of efforts to safeguard nature (Watson *et al*., 2014). Protected areas are the most common intervention, with over 300,000 designated to date (UNEP‐WCMC & IUCN, 2024). In addition, over 6,000 ‘other effective area‐based conservation measures’ (OECMs)’ have been recognised, comprising sites outside protected areas that are governed and managed in ways that achieve positive and sustained long‐term biodiversity outcomes (UNEP‐WCMC & IUCN, 2024; Jonas, Wood & Woodley, 2024). Many sites qualifying as OECMs have yet to be documented, as the definition of OECMs was adopted as recently as 2018, so only a small proportion of countries have formally recognised OECMs to date. Together, protected areas and OECMs cover over 17% of terrestrial and inland waters and 8.4% of marine and coastal areas (UNEP‐WCMC & IUCN, 2024).

Protected and conserved areas can successfully conserve biodiversity globally only if they are located strategically and managed effectively (Visconti *et al*., 2019a,b), which requires knowledge of the locations of sites that are of greatest importance for biodiversity. Efforts to identify a global network of such sites began in the late 1970s with the identification of Important Bird Areas (IBAs) by BirdLife International (Donald *et al*., 2019), followed by a proliferation of analogous approaches for other taxa and biodiversity features. To harmonise methods and produce a unified global inventory, a global standard with criteria for the identification of Key Biodiversity Areas (KBAs) was published in 2016 (IUCN, 2016), followed shortly afterwards by the establishment of the KBA Partnership, launch of the KBA website, and development of the World Database of KBAs (KBA Partnership, 2024a,b, 2025b).

As well as informing protected area identification and site‐based conservation efforts, KBAs are also very relevant to other aspects of nature conservation. They relate to multiple targets in the Kunming–Montreal Global Biodiversity Framework, which has been adopted by the world's governments to set commitments for addressing the loss and degradation of nature. For example, KBAs inform efforts in relation to spatial planning (Target 1), restoration of degraded ecosystems (Target 2), halting extinctions (Target 4), tackling invasive alien species (Target 6), pollution (Target 7), climate change (Target 8), and knowledge‐sharing (Target 21) as well as Target 3 on protected areas and OECMs (Plumptre *et al*. 2025a).

Consequently, KBAs have become widely recognised and used in policy and practice, including by multilateral environmental agreements, businesses, financial institutions and donors, and by governments and non‐governmental organisations. They also increasingly inform research in conservation science.

Nearly a decade after the launch of the global KBA Standard, Partnership and website, it is timely to review progress and to assess the impact of KBAs. Here we introduce the development of the KBA concept and data set (Brooks & Langhammer, 2025), summarise the KBA criteria and identification process, describe the extent and characteristics of the current KBA network (including threats and conservation interventions in place), review the extent and range of policy applications of KBAs, and explore future priorities.

## II. METHODS

We reviewed the published and grey literature on KBAs and analysed data from the World Database of KBAs as presented on the KBA website (https://www.keybiodiversityareas.org). Most analyses were based on all KBAs confirmed by September 2024, including 16,510 published KBAs (KBA Partnership, 2024a) and a further 86 for which details were withheld from publication because they support sensitive species for which the publication of distributional information could put them at risk (e.g. from capture for the pet or horticultural trade).

We analysed the prevalence of different habitats in KBAs using data from 11,032 KBAs (67%) for which habitats have been documented against the IUCN habitats classification scheme (https://www.iucnredlist.org/resources/habitat-classification-scheme). We also analysed the extent of KBAs (according to spatial analysis of their boundaries) and the KBA criteria (Table 1) under which each site qualified.

**Table 1:** Criteria for the identification of Key Biodiversity Areas (KBAs). Source: IUCN (2016). CR, Critically Endangered; EN, Endangered; VU, Vulnerable.

A. Threatened biodiversity	
A1. Threatened species	Site regularly holds one or more of the following: ≥0.5% of the global population size AND ≥5 reproductive units of a CR or EN species; ≥1% of the global population size AND ≥ 10 reproductive units of a VU species; ≥0.1% of the global population size AND ≥5 reproductive units of a species assessed as CR or EN due only to population size reduction in the past or present; ≥0.2% of the global population size AND ≥ 10 reproductive units of a species assessed as VU due only to population size reduction in the past or present; Effectively the entire global population size of a CR or EN species.
A2. Threatened ecosystem types	Site holds one or more of the following: ≥5% of the global extent of a globally CR or EN ecosystem type; ≥10% of the global extent of a globally VU ecosystem type.
B. Geographically restricted biodiversity	
B1. Individual geographically restricted species	Site regularly holds ≥10% of the global population size AND ≥ 10 reproductive units of a species.
B2. Co‐occurring geographically restricted species	Site regularly holds ≥1% of the global population size of each of a number of restricted‐range species in a taxonomic group, determined as either ≥2 species OR 0.02% of the global number of species in the taxonomic group, whichever is larger.
B3. Geographically restricted assemblages	Site regularly holds one or more of the following: ≥0.5% of the global population size of each of a number of ecoregion‐restricted species within a taxonomic group, determined as either ≥5 species OR 10% of the species restricted to the ecoregion, whichever is larger; ≥5 reproductive units of ≥5 bioregion‐restricted species OR ≥5 reproductive units of 30% of the bioregion‐restricted species known from the country, whichever is larger, within a taxonomic group; c Part of the globally most important 5% of occupied habitat for each of ≥5 species within a taxonomic group.
B4. Geographically restricted ecosystem types	Site holds ≥20% of the global extent of an ecosystem type.
C. Ecological integrity	
	Site is one of ≤2 per ecoregion characterised by wholly intact ecological communities, comprising the composition and abundance of native species and their interactions.
D. Biological processes	
D1. Demographic aggregations	Site predictably holds one or more of the following: An aggregation representing ≥1% of the global population size of a species, over a season, and during one or more key stages of its life cycle; A number of mature individuals that ranks the site among the largest 10 aggregations known for the species.
D2. Ecological refugia	Site supports ≥10% of the global population size of one or more species during periods of environmental stress, for which historical evidence shows that it has served as a refugium in the past and for which there is evidence to suggest it would continue to do so in the foreseeable future.
D3. Recruitment sources	Site predictably produces propagules, larvae, or juveniles that maintain ≥10% of the global population size of a species.
E. Irreplaceability through quantitative analysis	
	Site has a level of irreplaceability of ≥0.90 (on a 0–1 scale), measured by quantitative spatial analysis, and is characterised by the regular presence of species with ≥10 reproductive units known to occur (or ≥5 units for EN or CR species).

We extracted data on pressures from the most recent monitoring assessments for the subset of KBAs that qualify for birds (IBAs) as these are currently the only KBAs with monitoring data. We excluded assessments where pressures were not assessed, those assessed prior to 2006, and those with timing set as ‘Past (and unlikely to return) and no longer limiting’. This left a data set of 11,533 records for 3,867 KBAs in 163 countries or territories. Following KBA Partnership (2025a), the overall level of pressure to each site was scored as Very high, High, Medium or Low. We calculated the number and percentage of these sites that received each score, and the number for which different threats (classified following the hierarchical IUCN threats classification scheme: https://www.iucnredlist.org/resources/threat-classification-scheme) impact the species for which the site has been identified as internationally significant (directly, or *via* effects on the ecosystems they depend upon). We also calculated the number of sites impacted by each threat at level 1 in the IUCN threats classification scheme where the impact was scored as Very high, High, Medium or Low (based on scores for scope and severity of the threat, following KBA Partnership, 2025a). For the most frequent threat types, we also examined the frequency of threats at level 2 in the scheme. We also summarised statistics on levels and trends of potential threats to KBAs extracted from the KBA monitoring dashboard (http://bit.ly/42i3MLs), which presents the results of overlays between KBA boundaries and various spatial layers derived from satellite remote sensing (see details and methods in the Dashboard Guide at http://bit.ly/42i3MLs).

We analysed the conservation responses that have been documented by assessors at KBAs identified for birds (IBAs) over the past 20 years (2006–2025) using a standardised monitoring protocol (BirdLife International, 2006), including 2,995 KBAs with documentation on the adequacy of management planning and 2,739 KBAs with documentation on adequacy of conservation actions being implemented. We assessed which KBAs overlapped with protected areas that had management effectiveness assessments documented in the October 2025 version of the Global Database on Protected Area Management Effectiveness (https://www.protectedplanet.net/en/thematic-areas/protected-areas-management-effectiveness-pame). For cases where ≥75% of the area of both the KBA and the protected area overlapped, we determined which management effectiveness assessment system had been applied.

We quantified the contributions of terrestrial KBAs to climate change mitigation by rasterizing KBA digital boundaries (BirdLife International, 2025a), overlapping with a layer of vulnerable carbon (defined as carbon that would be lost during a typical land conversion event; Noon *et al*., 2022) and calculating the proportion of total vulnerable carbon that lies within KBAs.

We analysed the coverage of KBAs by protected areas and OECMs following the methods used to calculate Sustainable Development Goal indicators 14.5.1, 15.1.2 and 15.4.1 (United Nations, 2025). We classified coverage as complete (≥98%), partial (2–98%) or none (<2%), and defined KBAs as marine, terrestrial, freshwater and mountain (following United Nations, 2025). We calculated the change in coverage of IBAs by Special Protection Areas (SPAs) designated under the EU Birds Directive from 2013 to 2023 using the most recent archived Natura2000 data sets from the European Environment Agency Datahub (EEA, 2025) and IBA polygon data sets for these time points.

Following the adoption of the Kunming–Montreal Global Biodiversity Framework in 2022, parties to the Convention on Biological Diversity (CBD) were required to update their National Biodiversity Strategy and Action Plans (NBSAPs). We reviewed 47 NBSAPs produced by March 2025 as well as an additional 43 countries that had submitted their targets to the online reporting tool (https://www.cbd.int/nbsap). We identified those that referenced KBAs in either the NBSAP or the online reporting tool. We also identified those that specifically set national targets that include KBAs.

## III. DEVELOPMENT OF THE KBA CONCEPT

While the roots of the KBA concept can be traced back to the early 1970s (Brooks & Langhammer, 2025), the approach in its modern form dates to 1979, when the International Council for Bird Preservation (now BirdLife International) and the International Waterfowl and Wetlands Research Bureau (now Wetlands International) commenced identification of sites that might be considered under the newly adopted Birds Directive of the European Council (which requires Member States to designate special protection areas). This led to publication of an inventory of nearly 700 Important Bird Areas (IBAs, subsequently renamed Important Bird and Biodiversity Areas; BirdLife International, 2014) in the Member States of the European Community (Osieck & Mörzer Bruyns, 1981; Donald *et al*., 2019). Subsequent efforts led to the identification of IBAs across the rest of the world, with over 13,000 sites identified and documented to date (Donald *et al*., 2019; BirdLife International, 2025b).

The success of the IBA programme and its policy impact (Waliczky *et al*., 2019) inspired several analogous initiatives, including Important Fungus Areas (Evans, Marren & Harper, 2001), Important Plant Areas (Plantlife International, 2004) and Prime Butterfly Areas (van Swaay & Warren, 2006). The concept of KBAs as a threshold‐based approach to identification of important sites across multiple taxonomic groups emerged in the early 2000s, with criteria described by Langhammer *et al*. (2007) and applied in freshwater (Holland, Darwall & Smith, 2012), marine (Edgar *et al*., 2008) and terrestrial systems (Eken *et al*., 2004). In addition, the Alliance for Zero Extinction (AZE) identified sites supporting the last remaining population of one or more Critically Endangered or Endangered species. (Ricketts *et al*., 2005).

The development of multiple schemes and data sets of (often overlapping) important sites caused a degree of confusion among end‐users, prompting efforts to develop a unified approach and consolidate criteria and methods (Brooks & Langhammer, 2025). A Joint Task Force on Biodiversity and Protected Areas, established by the Species Survival Commission and the World Commission on Protected Areas, coordinated an extensive consultative process during 2011–2016 with experts in conservation organisations, academia, governments, donors and the private sector to consolidate methods for identifying KBAs as sites that contribute significantly to the global persistence of biodiversity (Brooks & Langhammer, 2025). This led to the publication of *A Global Standard for the Identification of Key Biodiversity Areas* in 2016 (IUCN, 2016).

Following the launch of the global KBA Standard, the KBA Partnership was established in 2016, comprising BirdLife International, IUCN, the Amphibian Survival Alliance, Conservation International, the Critical Ecosystem Partnership Fund, Global Environment Facility, Global Wildlife Conservation (subsequently renamed Re:wild), NatureServe, Royal Society for the Protection of Birds, World Wildlife Fund, and the Wildlife Conservation Society. Subsequently, the American Bird Conservancy and Rainforest Trust joined the Partnership. These 13 organisations jointly promote the identification, documentation, monitoring, conservation and safeguarding of KBAs, aiming to enhance global conservation efforts by adopting a common currency for internationally important sites for biodiversity, and promoting the direction of scarce resources to the most important places for nature. The Partnership appointed a KBA Secretariat to coordinate the KBA initiative, support KBA identification in countries worldwide, and promote conservation of KBAs (Langhammer, Butchart & Brooks, 2018).

## IV. KBA CRITERIA AND DELINEATION

Under the global KBA Standard, sites qualify as KBAs if they meet one or more of 11 criteria, clustered into five higher level categories: threatened biodiversity, geographically restricted biodiversity, ecological integrity, biological processes, and irreplaceability (Table 1). The KBA criteria can be applied to species and ecosystems in terrestrial, inland water, marine and subterranean environments, and may be applied across all taxonomic groups (although their application to micro‐organisms has yet to be tested).

The KBA criteria contain quantitative thresholds (Table 1), for example, relating to the proportion of the global population of a species or global extent of an ecosystem supported by a site. This ensures that identification is objective, repeatable and transparent, and that KBAs are comparable among countries and regions across the world (IUCN, 2016; Plumptre *et al*., 2024a, 2025a).

Each KBA is delineated as a ‘site’: a geographical area on land and/or in water with clearly defined ecological, physical, administrative or management boundaries that is actually or potentially manageable as a single unit (e.g. a protected area; IUCN, 2016). The identification of a site as a KBA indicates that it should be managed in ways that ensure the persistence of the biodiversity elements for which it is important. The form of this management is not prescribed: some KBAs are formally designated as protected areas, others are recognised OECMs, some are managed by local communities or individual landowners, and some have no management taking place (IUCN, 2016; Smith *et al*., 2018; KBA Standards and Appeals Committee, 2022). While some are already effectively managed, many need additional or expanded actions to conserve their biodiversity effectively.

## V. KBA IDENTIFICATION

**Fig. 1:**
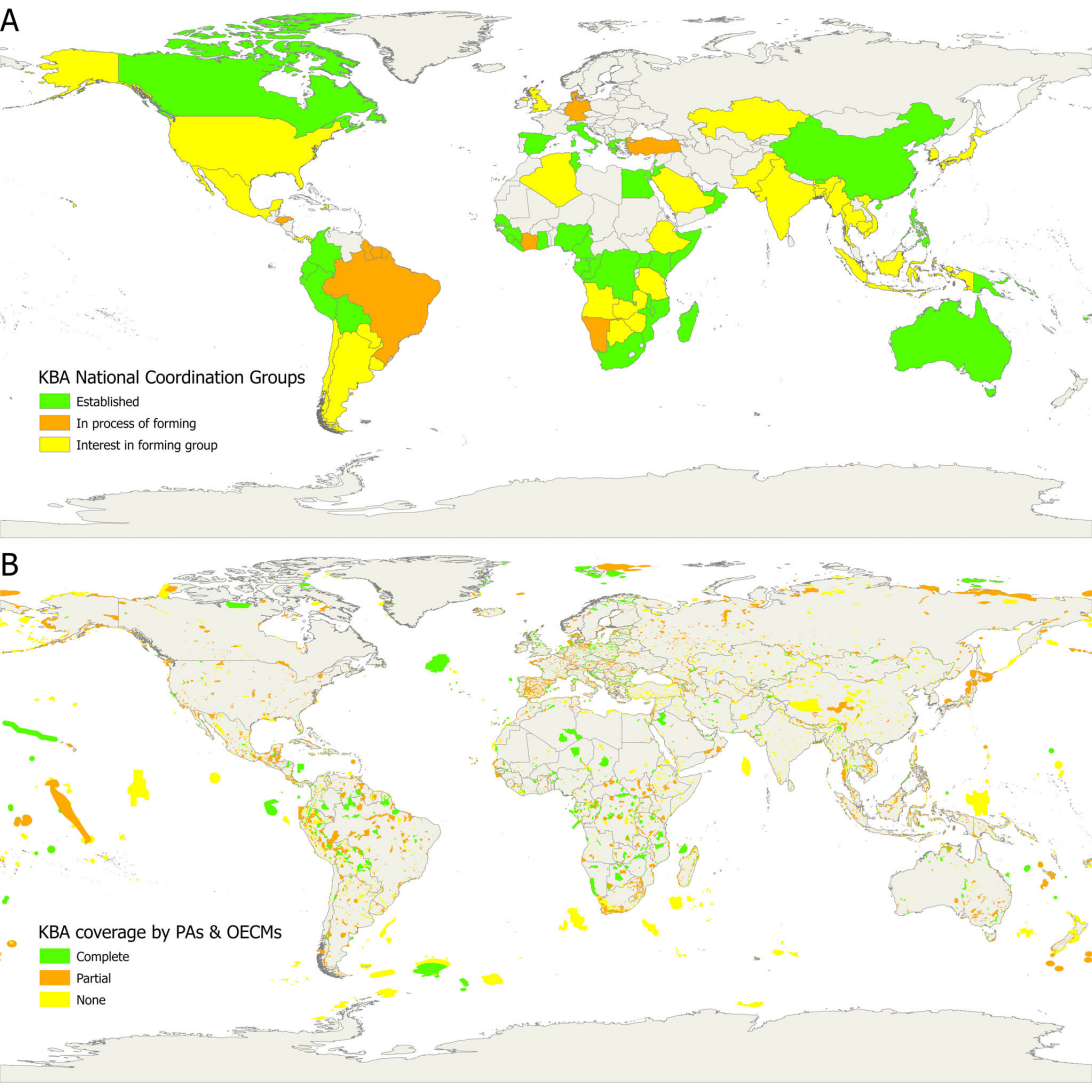
(A) The status of Key Biodiversity Area (KBA) National Coordination Groups in different countries, highlighting those that have been established (green, 41 countries), are in the process of establishing (orange, 12 countries), or where one or more institutions have indicated an interest in forming such a group (yellow, 28 countries). (B) The global KBA network, with the coverage of sites by protected areas (PAs) or other effective area‐based conservation measures (OECMs) shown as complete (green; 3,305 KBAs), partial (orange; 6,749 KBAs) or none (yellow; 6,141 KBAs).

KBAs can be proposed by anyone, but KBA assessments are typically developed by KBA National Coordination Groups (NCGs) composed of individuals and representatives from government, non‐governmental organisations, scientific institutions (including universities, herbaria and museums), and (where relevant) representatives of Indigenous Peoples' organisations. Such groups have now been established in 40 countries, and stakeholders in 39 additional countries are currently establishing KBA NCGs or are seeking the resources to do so (Fig. 1A).

These groups help to organise KBA identification, re‐assessment (when an existing KBA is re‐evaluated against the KBA criteria), monitoring and conservation at a national level and support the recognition of sites in national planning, policy and legislation (KBA Secretariat, 2024). They review ‘proposals’ (which compile relevant information and data justifying why a site qualifies as a KBA) for sites within their country prior to submission through the World Database of KBAs. Following review, proposals are then validated and confirmed by the KBA Secretariat and published on the KBA website (www.keybiodiversityareas.org) (Plumptre *et al*., 2025a).

This process ensures that the KBA criteria are correctly applied and KBA assessments are adequately documented so that users can be confident in the validity of the sites. Where no KBA National Coordination Group exists, individual proposers can submit proposals directly (KBA Secretariat, 2024). To support correct application of the KBA criteria, detailed guidelines (KBA Standards and Appeals Committee, 2022), tools (e.g. Beal *et al*., 2021; Spiliopoulou *et al*., 2024; BirdLife International, 2024) and training materials (https://www.conservationtraining.org/course/index.php?categoryid=150) have been developed in multiple languages, with over 1,500 people trained to date. The identification and delineation of KBAs is open to challenge, with an appeals process established for disputes over whether a site meets the KBA criteria.

## VI. DEVELOPMENT OF THE KBA DATA SET

When the KBA initiative was launched in 2016, a decision was taken not to discard the thousands of important sites identified up to that point, given their considerable recognition and widespread application by stakeholders across the conservation sector, including governments and businesses. Instead, sites identified under criteria that were most closely related to those in the global KBA Standard were incorporated into the KBA data set with the aim of reassessing them against the global KBA criteria in the subsequent years (IUCN, 2016). These included IBAs, AZE sites, and KBAs identified using the criteria outlined in Langhammer *et al*. (2007).

In 2018, BirdLife International analysed data on the 13,513 IBAs within this set of sites, demonstrating that 4,522 (33%) met global KBA criteria. These, together with more than 1,000 sites identified by the Alliance for Zero Extinction (for which the criteria match KBA criterion A1e) were classified as Global KBAs in the World Database of KBAs. A further 4,192 sites (31%) were classified as Regional KBAs where the available data showed that they did not meet the global KBA criteria but continued to meet previously established (i.e. IBA) criteria and thresholds, as recommended in the global KBA Standard (IUCN, 2016). Finally, 4,799 sites (36%) were classified as ‘Global/ Regional to be determined’ because there were insufficient data to determine whether they met global KBA criteria. Since then, 467 of these sites have been reassessed, with 258 shown to meet global KBA criteria and hence reclassified as Global, 68 delisted because they did not meet global KBA criteria and no longer qualified as IBAs (for example, because their qualifying species are no longer classified as threatened on the IUCN Red List), and 141 superseded, i.e. replaced by other sites with different boundaries. Reassessment of the 4,332 sites derived from IBAs still classified as ‘Global/ Regional to be determined’ is ongoing (as is the case for 2,200 that were solely identified under the previous KBA criteria described by Langhammer *et al*., 2007). A recent analysis of IBAs and KBAs focused on 11 countries (mostly highly biodiverse and tropical) in which relatively comprehensive or systematic KBA assessments have been carried out (i.e. considering all KBA criteria and taxonomic groups for which suitable data are available). This found that 87% of the area of KBAs qualifying for birds (i.e. IBAs) before the assessment was contained within global KBAs following the comprehensive assessment (Plumptre *et al*., 2025b). This suggests that many IBAs classified as Regional KBAs for birds or with a KBA classification of ‘Global/Regional to be determined’ will likely qualify as global KBAs (for birds or other taxa) following comprehensive assessments.

Since 2016 when the global KBA Standard was published, 2,085 sites in 132 countries have been assessed using the new criteria. Relatively comprehensive assessments (albeit mostly focused on terrestrial and freshwater environments) have been carried out in 11 countries: Bolivia, Colombia, Democratic Republic of Congo, Ecuador, Gabon, Mozambique, Peru, Republic of Congo, South Africa, Uganda, and United Arab Emirates. Across these 11 countries, application of the global KBA Standard increased the mean number of KBAs per country by 70% and the mean total extent of KBAs per country by 164%. The mean proportion of each country covered by KBAs increased from 13.9 to 24.6% on land, 17.9 to 27.3% for fresh standing waters, 13.3 to 23.6% for river and stream length, and 1.6 to 10.3% of marine territorial areas (Plumptre *et al*., 2025b).

In Canada, application of the global KBA Standard to identify sites of significance for the global persistence of biodiversity has been accompanied by the identification of KBAs of national significance (which are not included in the World Database of KBAs or the KBA website) (KBA Canada, 2025). A total of 456 such sites were identified by calculating KBA thresholds using national instead of global populations and extents, considering national instead of global extinction risk categories, and including KBAs for nationally recognised subspecies, populations and varieties. These nationally significant KBAs increased the number of important sites in the country by 126% from 362 to 818, the total extent of important sites by 28% from 623,131 km^2^ to 794,598 km^2^ and the coverage of Canada's area from 6.2 to 8.0% (KBA Canada, 2025).

## VII. CHARACTERISTICS OF THE GLOBAL KBA NETWORK

**Fig. 2:**
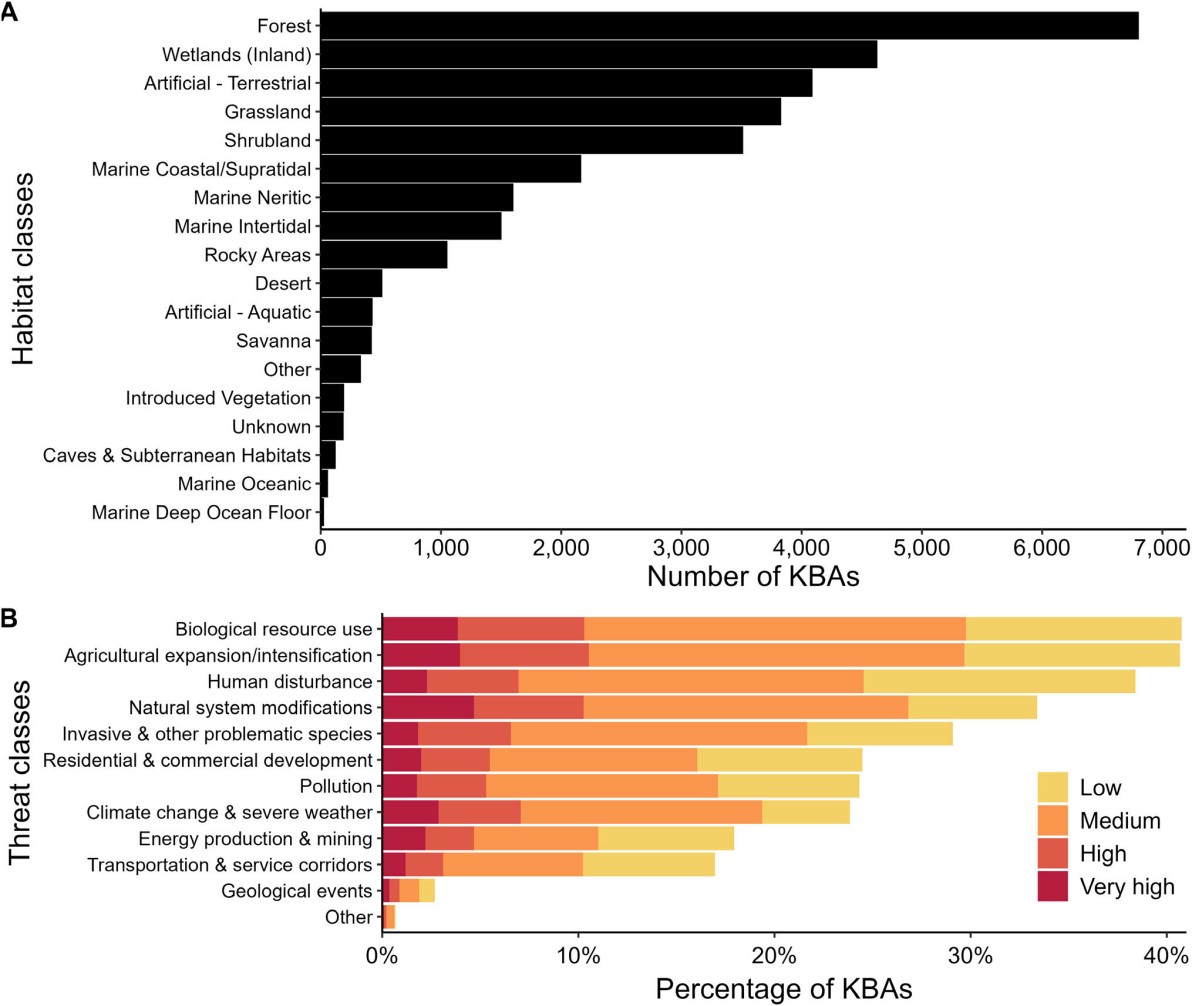
(A) Number of Key Biodiversity Areas (KBAs) supporting different habitat types (*N* = 11,062 KBAs for which habitats have been documented against the IUCN habitats classification scheme, representing 67% of all KBAs). (B) Percentage of KBAs impacted by different threats (with classes defined at level 1 in the IUCN threats classification scheme, and threat impact scored from low to very high; *N* = 11,533 records of 3,867 KBAs in 163 countries or territories, representing 23% of all KBAs).

A total of 16,596 KBAs covering 22.1 million km^2^ have been documented and confirmed in the World Database of KBAs and published on the KBA website as of September 2024 (Fig. 1B, Table 2; details for 86 sensitive sites are not published). All of the world's countries/territories and the High Seas have at least one confirmed KBA, with the exception of Tuvalu, Vatican City, San Marino and Monaco (four of the five smallest countries in the world). Comparing regions, Europe holds the largest number of KBAs identified to date (29% of all KBAs), reflecting the long history of IBA identification, widespread application of regional IBA criteria, and smaller average size of sites in this region (Donald *et al*., 2019). Asia supports 21% of KBAs, Africa 13% and South America 10% (see Table 2). Most KBAs (96%) are terrestrial, while 29% are marine, 26% are freshwater, and <1% (25 KBAs) are subterranean (many KBAs span more than one system). Most KBAs are found in forest (61% of the 11,062 KBAs that have habitat types documented), inland wetlands (42%), artificial terrestrial (37%) and/or grassland habitats (35%) (Fig. 2, note that only 67% of KBAs have habitats documented, and most contain multiple habitats).

**Table 2:** Number of Key Biodiversity Areas (KBAs) by system, KBA classification and region. Totals date from September 2024. Note that some coastal sites are classified as both terrestrial and marine.

Region	No. (area in km^2^) of KBAs (total)	No. (area in km^2^) of KBAs (terrestrial/ freshwater)	No. (area in km^2^) of KBAs (marine)	% terrestrial/ freshwater coverage	% marine coverage	No. (%) Global	No. (%) Regional	No. (%) Global/ Regional to be determined
Africa	2,155 (3,453,109)	1,881 (3,115,789)	353 (468,266)	10.6	1.7	935 (43)	119 (6)	1,105 (51)
Antarctica	246 (494,580)	230 (8,404)	245 (494,580)	0.05	8.2	114 (46)	118 (48)	14 (6)
Asia	3,433 (3,550,287)	3,335 (3,212,634)	768 (1,125,955)	9.1	2.5	555 (16)	260 (8)	2,618 (76)
Australasia	759 (1,453,716)	710 (564,068)	380 (999,024)	6.1	5.0	503 (66)	11 (1)	245 (32)
Caribbean	430 (62,419)	418 (62,299)	233 (36,965)	19.7	0.51	191 (44)	51 (12)	188 (44)
Central America	200 (192,118)	194 (184,351)	50 (57,248)	20.9	1.8	125 (63)	12 (6)	63 (32)
Central Asia	765 (623,105)	765 (623,105)	50 (41,824)	6.0	1.5	264 (35)	200 (26)	301 (39)
Europe	4,862 (1,572,396)	4,583 (1,390,307)	1,343 (539,657)	10.0	3.1	1,065 (22)	2,896 (60)	901 (19)
High Seas	63 (2,728,924)	–	63 (2,728,924)	–	1.3	59 (94)	2 (3)	2 (3)
Middle East	505 (397,430)	416 (370,924)	108 (4,4871)	7.2	1.5	146 (29)	119 (24)	240 (48)
North America	1265 (1,980,375)	1167 (1,658,783)	574 (1,224,254)	3.5	6.1	652 (52)	228 (18)	385 (30)
Oceania	320 (2,279,756)	235 (192,924)	254 (2,271,580)	34.9	8.0	158 (49)	40 (13)	122 (38)
South America	1,593 (3,329,995)	1,556 (3,058,738)	265 (638,552)	15.8	3.7	1,178 (74)	67 (4)	348 (22)
Global	16,596 (22,118,212)	15,490 (14,442,328)	4,686 (10,671,700)	8.6 (9.4)^†^	4.3* (4.2)*,^†^	5,941 (36)	4,123 (25)	6,532 (39)

* Excludes High Seas.

^†^ Excludes Antarctica.

**Fig. 3:**
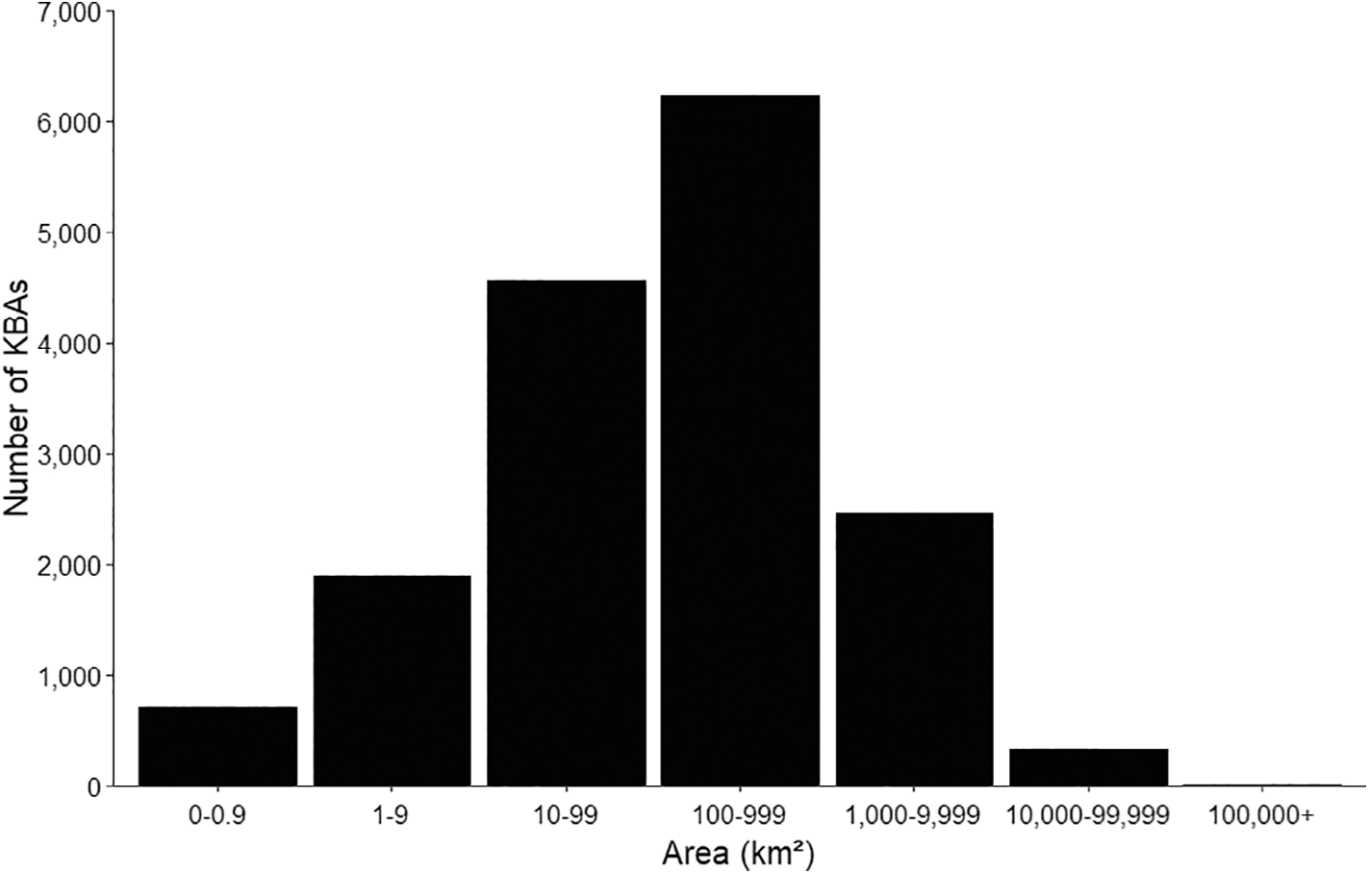
Frequency distribution of Key Biodiversity Areas (KBAs) of different extents.

Currently, 5,941 sites (36%) are classified as Global KBAs, 4,123 (25%) as Regional KBAs, and 6,532 (39%) have a classification as ‘Global/Regional to be determined’ (almost half of which are located in Asia, owing to less complete documentation of the biodiversity importance of these sites), with work ongoing to resolve these. The spatial extent of KBAs varies from 0.001 km^2^ to 712,457 km^2^, with a mean of 1,364 km^2^, a median of 141 km^2^, and 38% of sites falling in the range 100–999 km^2^ (Fig. 3). The largest KBAs are marine (including 18 of the 20 largest KBAs), and the mean area of solely marine (i.e. excluding coastal) sites (13,287 km^2^, *N* = 556) is significantly greater than that of solely terrestrial sites (1,010 km^2^, *N* = 11,265; Welch's *t* = 5.91, d.f. = 553.5, *P* < 0.0001). Hence, while there are fewer marine than terrestrial sites, they comprise 48% of the total area of KBAs.

**Fig. 4:**
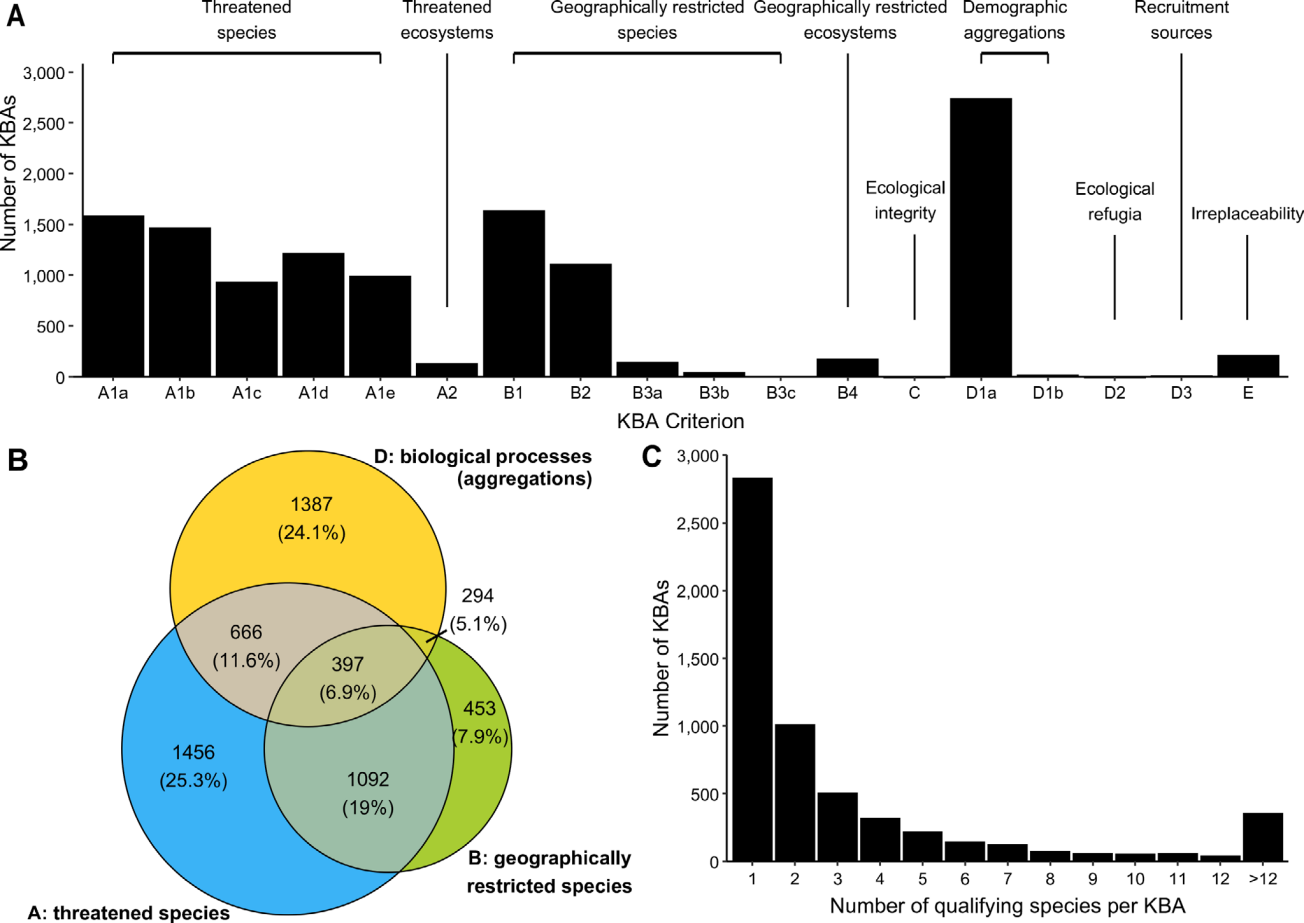
(A) Number of Key Biodiversity Areas (KBAs) qualifying under different KBA criteria (showing the relevant subdivisions of criteria A1, B3 and D1; see Table 1). (B) Number of KBAs qualifying under different species‐related KBA criteria and overlaps between these subsets (percentages indicate the proportion of all KBAs; *N* = 5,605 KBAs (34% of all sites) that have sufficient data to indicate under which global KBA criteria they qualify; reassessment of other KBAs is ongoing). (C) Number of KBAs qualifying for different numbers of species under KBA criteria A, B and D.

Many Global KBAs (43%), qualify under multiple criteria (Fig. 4). Most (63%) of these sites qualify under one or more of the KBA criteria relating to globally threatened species (A1a–e), and almost half (48%) qualify under the criteria relating to biological processes (mostly demographic aggregations; D1a), while 39% qualify under criteria relating to geographically restricted species (B1–3).

To date, 3.0% of KBAs qualify under criterion A2 for threatened ecosystem types or B4 for geographically restricted ecosystem types. However, it should be noted that many countries lack systematic ecosystem assessments, such as under the IUCN Red List of Ecosystems (Keith *et al*., 2013), and that application of KBA criteria A2 and B4 requires ecosystems to be mapped using a consistent classification over their entire extent (IUCN, 2016), posing challenges for KBA assessors. Similarly, just one KBA (Nouabalé‐Ndoki National Park in Congo) currently qualifies owing to its globally significant ecological integrity (under criterion C), because guidelines on the application of this criterion have only recently been developed. Only 205 KBAs (3.6%, all in South Africa) qualify for their high irreplaceability measured by quantitative spatial analysis (criterion E). As more countries update their inventories using the global KBA Standard and as relevant data become more widely available, we expect the proportion of KBAs qualifying under the KBA criteria relating to ecosystems, ecological integrity and irreplaceability to increase substantially (although there is no *a priori* reason to expect parity in the numbers of sites qualifying under different KBA criteria). The number of sites qualifying for taxonomic groups that are not well represented in the current data set is also likely to grow.

**Fig. 5:**
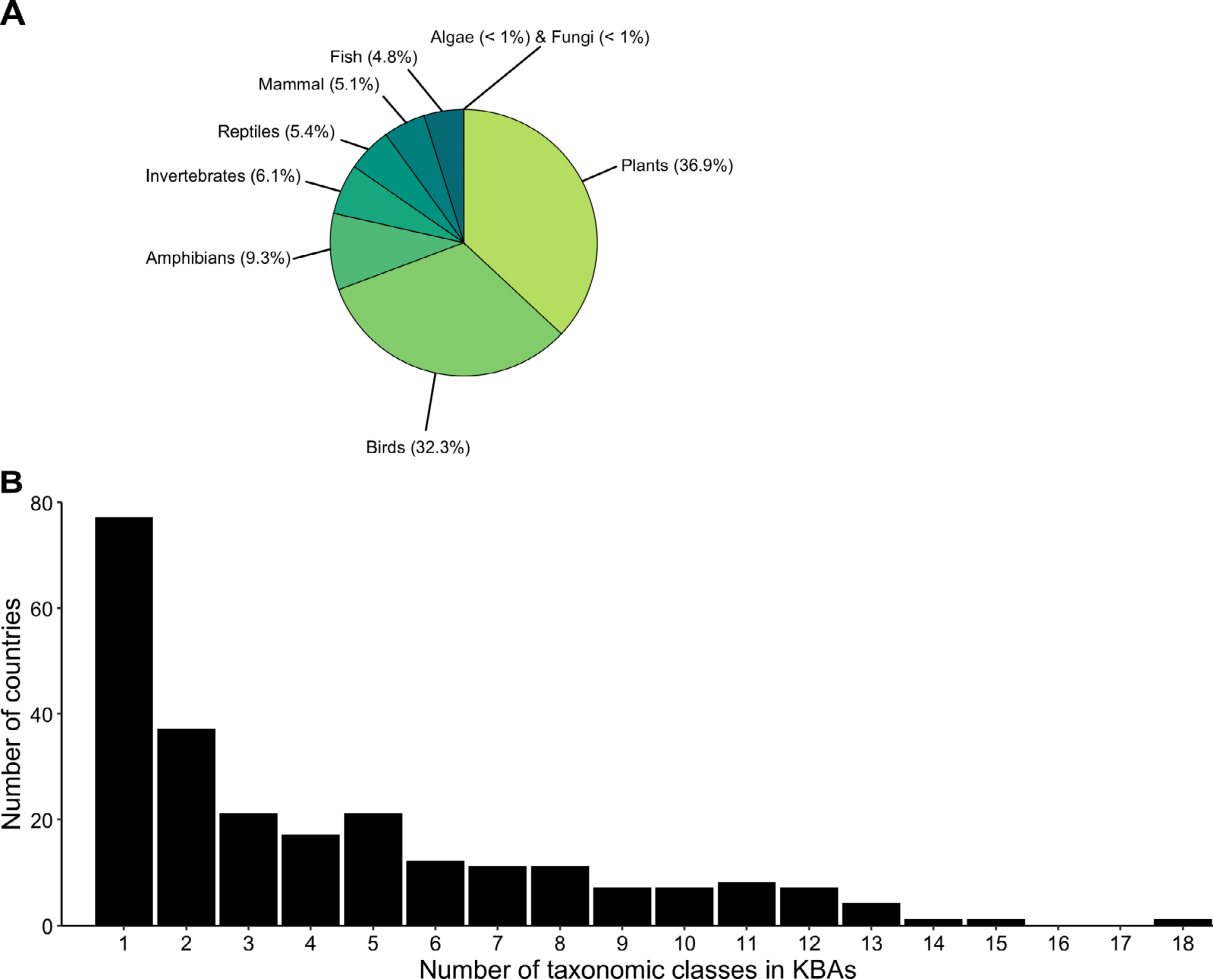
(A) Taxonomic composition of species for which Key Biodiversity Areas (KBAs) have been identified (*N* = 18,365 species). (B) Number of countries with KBAs identified for different numbers of taxonomic classes (*N* = 15,933 KBAs).

The number of species for which each site qualifies as a KBA ranges from 1 to 320, with 49% of sites (2,832) qualifying for one species and 81% (4,656) qualifying for four or fewer species (Fig. 4). To date, KBAs have been identified as important for a total of 18,365 qualifying species, mostly comprising plants (37%, predominantly Magnoliopsida) and birds (32%), followed by amphibians (9%), invertebrates (6%, mostly Insecta, Gastropoda, Anthozoa and Malacostraca) reptiles (5%), mammals (5%), and ray‐finned fishes (5%; Fig. 5).

## VIII. THREATS TO KBAS

For 3,867 KBAs in 163 countries and territories for which threats have been assessed in the last 20 years (23.3% of all KBAs), the most prevalent threats are biological resource use (impacting 40.8% of these sites), agricultural expansion and intensification (40.7%), human intrusions and disturbance (38.4%), and natural systems modifications (water management and fire; 33.4%, Fig. 2B). Among sites threatened by biological resource use, most are impacted by hunting/collecting of terrestrial animals (27.4% of all 3,867 sites assessed), logging and wood harvesting (13.7%), and fishing and harvesting aquatic resources (10.8%). Among sites threatened by agriculture, most are impacted by annual/perennial non‐timber crops (22.0% of all 3,867 sites assessed), and livestock farming and ranching (21.9%). Finally, among sites threatened by human intrusions and disturbance, most are impacted by recreational activities (29.0% of all sites) and work and other activities (11.5%). Often, there are interactions between threats (e.g. roads increase the risk of hunting through increasing ease of access). Most of the threats to KBAs were estimated as having medium (46.1% of 11,533 site–threat combinations) or low (30.0%) impact, with a relatively small proportion estimated to have very high (9.1%) or high impact (14.9%; Fig. 2B). Considering only threat records with very high, high or medium impact did not change the relative sequence of the most important threats, except for natural systems modifications (including dams/water management and fire/fire suppression) which then ranked higher than human intrusions and disturbance.

These and other threats have driven substantial deforestation in terrestrial KBAs, with sites losing 8.2% of their tree cover on average between 2001 and 2020, and the annual rate of tree cover loss increasing during this period (http://bit.ly/42i3MLs). Deforestation has been higher in KBAs with lower coverage by protected areas, at lower elevations, and with less steep slopes, higher human population density and in countries with lower *per capita* national Gross Domestic Product (GDP) (Tracewski *et al*., 2016). Over half (56%) of the 3.9 million km^2^ of forest in 6,844 KBAs identified for forest species has low or medium integrity according to the Forest Landscape Integrity Index (Grantham *et al*., 2020), with KBAs in Europe, Central America, and the Caribbean having the highest proportion of their forest classified as low or medium integrity. Forest integrity is proportionally lower in KBAs that are smaller, contain proportionally less forest, located far from other forested KBAs, and outside protected areas (Crowe *et al*., 2023a).

Approximately 30% of terrestrial KBAs face severe human impacts as measured using the human footprint index (Venter *et al*., 2016), with European KBAs having the highest average index levels (Yang *et al*., 2024). Human impacts have led to the halving of mean patch size and the tripling of the perimeter:area ratio for natural habitats in KBAs (Yang *et al*., 2024).

At least 80% of KBAs contain infrastructure, including for transport (airports, ports, roads and railways), energy (powerlines and powerplants), resource extraction (mines and oil and gas pipelines, wells, and facilities), urbanisation, and dams and reservoirs (Simkins *et al*., 2023a). Roads are most prevalent, occurring in 75% of KBAs. Data on potential future development of mines, oil and gas infrastructure and powerplants suggest that there will be considerable growth in their impact if planned infrastructure, claims and inactive concessions materialise, including for 22% of KBAs that currently contain no infrastructure (Simkins *et al*., 2023a). Another study found that more than 1,200 mining sites lie within terrestrial KBAs, with 29% of these involving minerals that are key to the energy transition (Whieldon *et al*., 2022).

KBAs are heavily impacted by light pollution, which has pervasive impacts on the physiology and behaviour of individual organisms (e.g. hatching of turtle nestlings, navigation of migrating birds), the abundance and distribution of species, and the structure and function of ecological communities (Karan, Saraswat & Anusha, 2023). Only 30% of KBAs have completely unpolluted night skies and 52% lie entirely under artificially bright skies, with light pollution affecting the greatest proportion of KBAs in Europe and the Middle East (Garrett, Donald & Gaston, 2019). Night light radiance in KBAs grew by 34% on average between 2014 and 2018 alone (http://bit.ly/42i3MLs). These patterns are linked to the density of human populations, which reached a mean of 96 people per km^2^ in KBAs in 2020 and grew by 1.2% during 2010–2020 (http://bit.ly/42i3MLs).

Climate change is having pervasive impacts on KBAs, and these impacts are projected to become increasingly severe. Studies to date have been most extensive for KBAs qualifying for birds (IBAs). These studies model species' distributions in relation to climatic variables, project their future distributions under different scenarios, and then assess the consequences for site networks. The results show that considerable turnover is projected in the qualifying species, i.e. some of the species for which a site currently qualifies as an IBA are projected to have no suitable climate (defined as climatic conditions falling within the variation occurring across the current distribution) within the site boundary in future, while other species that could qualify the site as an IBA in future are projected to colonise these sites (taking account of species' differing dispersal abilities). This turnover increases with time and is greater under more severe climate change scenarios (BirdLife International and National Audubon Society, 2015). Average turnover of IBA‐qualifying species within IBAs is predicted to be 17% by 2050 for birds in Central and South America and the Caribbean (Voskamp *et al*., 2021), 35–45% by 2085 for sub‐Saharan Africa (Hole *et al*., 2009), and 43% by 2085 for the Eastern Himalaya and Lower Mekong hotspots (Bagchi *et al*., 2013). Analogous work in West African protected areas suggests that mammals have similar projected turnover as birds, but rates will be higher for amphibians (Baker *et al*., 2015).

Nevertheless, research suggests that site networks as a whole will remain robust under climate change (at least for birds), with the vast majority of species retaining suitable climate in at least some of the sites in which they currently occur (Hole *et al*., 2009; Bagchi *et al*., 2013; Voskamp *et al*., 2021). While up to 66% of tropical forest KBAs have recently transitioned to novel below‐canopy temperature regimes, one‐third of KBAs provide refuge from novelty (Trew *et al*., 2024). Management strategies at individual KBAs will need to become more dynamic under climate change and consider each site in the context of the wider KBA network (Hole *et al*., 2011; Breiner *et al*., 2022). However, KBA conservation should remain an integral part of nature‐based solutions for climate change in order to safeguard biodiversity and benefit people (Butchart *et al*., 2021).

For KBAs qualifying for migratory species, threats to one or a small number of sites may have population‐level impacts if such sites support a substantial cumulative proportion of the population through the annual cycle. For example, recent loss of tidal mudflats in KBAs in the Yellow Sea is driving rapid declines in global populations of shorebirds that rely on these locations as stop‐over sites to refuel on migration (Studds *et al*., 2017).

## IX. ECOSYSTEM SERVICE VALUES OF KBAS

In addition to their significance for biodiversity, ecosystems within KBAs provide important contributions to people including climate mitigation, freshwater provision and purification, flood mitigation, coastal protection, tourism, and cultural identity. Globally, KBAs cover around 10% of land area but store nearly 15% of vulnerable carbon (i.e. carbon that would be lost during a typical land‐use conversion event; Noon *et al*., 2022). Regionally, a study in West Africa found that KBAs cover 7.4% of land but store 16.0% of vegetation carbon and sequester 17% of carbon per annum (Buchanan *et al*., 2021). KBAs in China have been shown to provide significant benefits in the form of carbon storage, nutrient regulation, and landscape aesthetics (Dong *et al*., 2024), while KBAs in Myanmar provide clean water and flood risk mitigation for vulnerable villages (Mandle *et al*., 2017). In Madagascar, KBAs provide water quality regulation which supports household use, crop irrigation, and hydropower, deliver protection from coastal flooding, and provide non‐timber forest products, carbon storage, and benefits from tourism (Neugarten *et al*., 2016).

Given their global significance for biodiversity, KBAs also provide irreplaceable opportunities for recreation and ecotourism (e.g. wildlife viewing; Neugarten *et al*., 2016; Hausmann *et al*., 2019). Conserving wildlife also supports cultural identity, spiritual inspiration, opportunities for learning and education, ecosystem engineering, trophic dynamics, pest control, provision of natural medicines and pharmaceuticals and pollination (Chaplin‐Kramer *et al*., 2025). The subset of KBAs qualifying as AZE sites also cover significant areas of human linguistic diversity (Larsen, Turner & Brooks, 2012), adding to their importance for the world's cultural heritage. An example of cultural identity linked to a KBA is the Sierra Nevada de Santa Marta National Natural Park in Colombia, for which one of the qualifying species is the starry night harlequin toad *Atelopus arsyecue*. This species is regarded as a guardian spirit by the Arhuaco Indigenous community, providing guidance on the optimal timing for agricultural activities and spiritual ceremonies.

Furthermore, conserving sites such as KBAs provides economic benefits. For example, a study of conservation and/or restoration benefits of 62 sites globally (including many KBAs) indicates that their value for ecosystem services outweighs their private value (such as potential profits from agriculture or logging) (Bradbury *et al*., 2021). A single wetland KBA in Myanmar provides benefits estimated at USD 22 million/year to local communities and downstream rice farms, in the form of climate regulation, recreation, flood protection, water provision, food and fodder, rice production, and biodiversity (Aung *et al*., 2021). Given the ecosystem services that KBAs provide to people, it is notable that the benefits of conserving biodiversity flow disproportionately to the world's poor, contributing to poverty reduction (Turner *et al*., 2012).

## X. CONSERVATION RESPONSES AT KBAS

Conservation responses have been monitored by assessors at more than 3,200 KBAs over the past 20 years using the IBA monitoring protocol (BirdLife International, 2006, 2014), generating scores that reflect the extent of designation, management planning and conservation actions being implemented. These assessments show that only 48.5% of 2,995 assessed KBAs have management plans, of which less than half are comprehensive and aim to maintain or improve the populations of qualifying species. Only 36.4% of 2,325 assessed KBAs with documentation on the adequacy of implementation have comprehensive or substantive conservation measures being implemented. Assessments of protected area management effectiveness using various evaluation systems have been compiled into the Global Database on Protected Area Management Effectiveness (https://www.protectedplanet.net/en/thematic‐areas/protected‐areas‐management‐effectiveness‐pame). Fewer than one in 10 KBAs (8.3%) has substantial overlap with a protected area that has had management effectiveness assessed according to this data set; of these, the most commonly applied method was the IBA monitoring protocol (43.5%), the Management Effectiveness Tracking Tool (25.9%) and the Common Standards Monitoring (15.7%, all in the UK).

### (1) Protected areas and OECMs

**Fig. 6:**
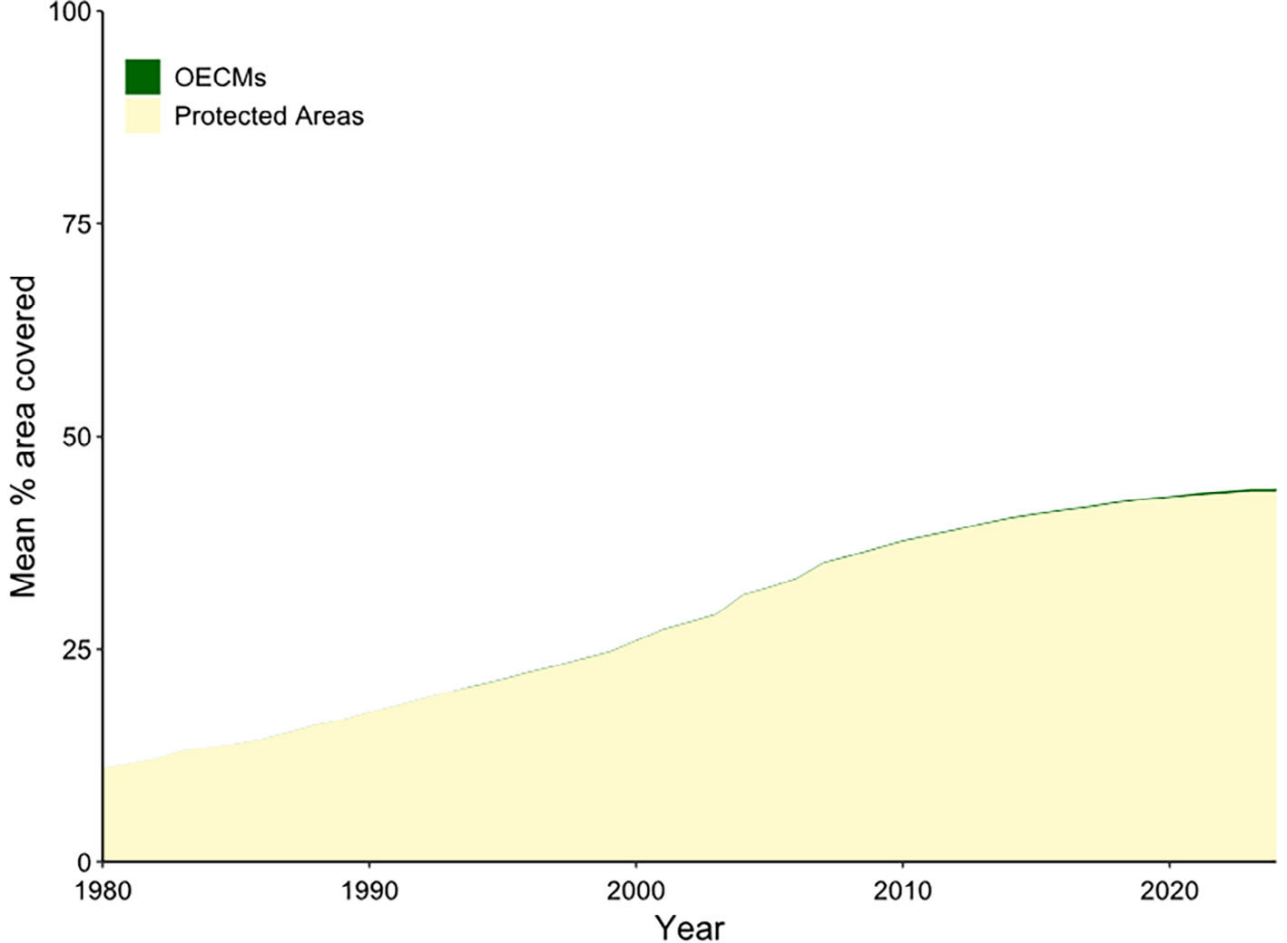
Coverage of Key Biodiversity Areas (KBAs) by protected areas and other effective area‐based conservation measures (OECMs).

At least 62% of KBAs overlap with protected areas and/or OECMs, and 20% of KBAs are completely covered (Fig. 1B). Europe and Africa have the highest proportion of KBAs completely covered (35 and 22%, respectively) while <10% of KBAs in Asia, Central Asia and the High Seas are completely covered. Since 2000, the mean percentage area of KBAs covered by protected areas and/or OECMs has increased from 25 to 44% (Fig. 6), with similar trends for terrestrial, marine and freshwater KBAs. However, the rate of increase has been slowing, and the proportion of all protected areas that are KBAs has been decreasing (Visconti *et al*., 2019a). KBAs in Europe and the Caribbean have experienced the greatest increase in mean percentage coverage (37 to 64% and 18 to 40% respectively since 2000). The smallest increases in coverage across the same time period were for KBAs in the High Seas (0–1.6%) and North America (27–35%).

### (2) KBA conservation by indigenous peoples and local communities

Many KBAs fall within lands that are managed by Indigenous Peoples, or to which they have legal rights. In 83 countries for which Indigenous Peoples' lands have been mapped, these overlap at least partly with 34% of the KBAs in these countries, covering 36% of the total extent of these KBAs, which equates to 28% of the total area of land in all KBAs globally (Simkins *et al*., 2023b). Given that 14% of the total area of land in KBAs globally fell within Indigenous Peoples' lands but outside formal protected areas, this suggests that Indigenous Peoples may play a potentially significant role in conserving KBAs. Furthermore, a matching analysis showed that tree cover loss in unprotected KBAs was lower inside than outside Indigenous Peoples' lands, although tree cover loss in KBAs was lowest in protected areas, regardless of whether they were inside or outside Indigenous Peoples' lands. This is likely because of government‐supported efforts to mitigate threats in many protected areas, whereas multiple land uses are often sanctioned within Indigenous Peoples' lands (Simkins *et al*., 2023b).

Whatever the legal status of KBAs, conservation and management that involves local people is likely to be more effective, sustainable and equitable (Dawson *et al*., 2021). Over 4,000 local conservation groups have been supported within or around at least 704 confirmed KBAs through efforts to conserve the subset of KBAs that are IBAs (BirdLife International, 2022). Members of such organisations typically live and work within or close to these sites, and their livelihoods often depend on ecosystem services delivered by them. Such groups are based on a common model of local, civil‐society engagement and empowerment, but vary in nature considerably between and within countries. Monitoring, mitigating threats, education and awareness raising, campaigning, and integrating conservation with local development are among the activities carried out (Waliczky *et al*., 2019). Local conservation groups at African KBAs often comprise pre‐existing community‐based organisations focused on linking conservation and sustainable management of natural resources to alleviate poverty. ‘IBA caretakers’ in Europe (individuals living locally at an IBA), ‘IBA adoption groups’ in the USA (bird clubs and other local community groups) and ‘KBA Guardians’ in Australia (individuals or local groups) provide stewardship of the site, implement active restoration interventions, coordinate citizen science monitoring efforts, and/or offer educational opportunities to support conservation. In Argentina, ‘Clubes de Observadores de Aves’ (birdwatching groups led by local environmentalists) are supporting conservation efforts at many IBAs, while local communities around IBAs in Lebanon are adopting a traditional system called *hima* that involves promoting the sustainable use of natural resources to manage and protect natural areas while improving livelihoods.

### (3) KBAs and systematic conservation planning

KBAs are a valuable input to Systematic Conservation Planning (SCP; Smith *et al*., 2018; Plumptre *et al*., 2024b). This discipline includes techniques for making objective decisions about where to prioritise conservation actions using a complementarity‐based optimisation algorithm. Outputs include maps with alternate options of where to conserve species, ecosystems or other biodiversity features to achieve targets set for their representation, while minimising the cost or area required. KBAs are a useful input into any SCP process because they identify sites that have globally significant populations of a species, extent of an ecosystem, outstanding ecological integrity or irreplaceability (Plumptre *et al*., 2024b). When developing conservation plans using SCP, users can ‘lock in’ KBAs (e.g. Plumptre *et al*., 2019) and then assess which additional areas will be needed to meet all the conservation targets (an approach that is often taken for existing protected areas). In other situations, particularly where the proportion of the global population of a species or global extent of an ecosystem supported by a KBA is very close to the relevant threshold defined in the KBA criteria, planners may prefer to take account of the recognised KBAs but not necessarily lock all of them into the final solution (Plumptre *et al*., 2024b), or set context‐specific targets for each KBA type (Smith *et al*., 2018). SCP techniques can also be applied when prioritizing among management actions for KBAs (Smith *et al*., 2018), while irreplaceability values from SCP spatial prioritisation analyses can be used to identify KBAs under KBA criterion E (IUCN, 2016).

## XI. POLICY APPLICATIONS

### (1) Informing designation of protected areas

While it is difficult to draw causal inferences, a number of lines of evidence and examples suggest that KBAs have had widespread impact in informing designation of protected areas. The strongest evidence comes from Europe, where data on KBAs for birds (IBAs) have influenced the designation of SPAs under the EU Birds Directive (Kukkala *et al*., 2016). The European Commission brought cases against several Member States to the European Court of Justice for failure to designate sufficient SPAs (including the Netherlands in 1998, Finland in 2000, France in 2002, Italy in 2003 and 2007, Spain in 2007 and 2016, Greece and Ireland in 2007, and Bulgaria in 2016; Waliczky *et al*., 2019). The court accepted the Commission's case that IBA inventories represented the best available evidence on locations qualifying as SPAs and required Member States to recognise these as SPAs. This led to an increase in the proportion of IBAs partly or completely covered by SPAs from 30% in 1989 to 54% in 1999 (BirdLife International, 2004), reaching 89% in 2023, while the proportion of the total extent of IBAs covered by SPAs has grown from 59% in 2013 to 67% in 2023. The coverage of IBAs by SPAs correlates positively with the year of accession to the EU, suggesting that the designation of SPA networks has been significantly informed by national IBA inventories in the more recently joined Member States in Central and Eastern Europe (Kukkala *et al*., 2016). Estonia, Bulgaria and Latvia all now have >90% of their IBA extent covered by SPAs (Kukkala *et al*., 2016; BirdLife International, 2018).

Elsewhere, there are numerous examples where identification of sites as KBAs (including IBAs and AZE sites) followed by advocacy by civil society organisations is likely to have informed the designation of protected areas. Self‐reported data by national BirdLife Partners suggested that 453 IBAs had been designated as formal protected areas (or existing protected areas had been expanded) during 2013–2022 at least partly as a consequence of their advocacy for the protection of these sites (BirdLife International, 2022). While the degree of influence no doubt varied considerably, there are multiple examples over recent decades where a strong case can be made that IBA/KBA identification directly influenced protected area designation or expansion. These include the declaration of Timor‐Leste's first national park (Nino Konis Santana National Park) in 2007 following systematic evaluation of the country's biodiversity sites in an IBA directory (Trainor *et al*., 2007; BirdLife International, 2007; Waliczky *et al*., 2019), and the declaration of Lo Go Xa Mat National Park in Vietnam in 2002 following its identification as an IBA (BirdLife International, 2004; Waliczky *et al*., 2019). In Colombia, 1,320 hectares of tropical forest were protected in 2024 by the local government (Corporación Autónoma Regional del Valle del Cauca) through a locally led process coordinated by the NGO Corporación Ambiental y Forestal del Pacífico following identification of the Enclave Seco del Río Dagua KBA and AZE site. Elsewhere in Colombia, the Alto Calima Regional Protected Area, which protects part of the Alto Calima Region KBA, was designated in 2024 through a collaborative process led by local and Indigenous communities, with technical support from Fundación Trópico and the regional environmental authority as part of the Conserva Aves initiative developed by BirdLife International, American Bird Conservancy, National Audubon Society, Birds Canada, and the Network of Latin American and Caribbean Environmental Funds (RedLAC).

KBAs have also informed protected area designation in the marine realm. A particularly strong example is the North Atlantic Current and Evlanov Sea basin (NACES) marine protected area (covering nearly 600,000 km^2^), which was identified as a KBA from an analysis of seabird tracking data for 21 species and designated in 2021 by the Oslo and Paris (OSPAR) Convention for the Protection of the Marine Environment of the North‐East Atlantic, following 5 years' of technical support and advocacy across the annual meeting cycle (Davies *et al*., 2021a,b). In South Georgia and the South Sandwich Islands, marine KBA identification led to the government expanding strict protection areas and temporal exclusion zones within the South Georgia and South Sandwich Islands Marine Protected Area (Handley *et al*., 2020, Belchier *et al*., 2022), one of the world's largest marine protected areas, covering 1.24 million km^2^.

### (2) Relevance of KBAs to the Convention on Biological Diversity (CBD)

KBAs are highly relevant for informing actions towards meeting the Goals and Targets of the Kunming–Montreal Global Biodiversity Framework. Most importantly, Target 3 in the framework commits parties to effective conservation of ‘areas of particular importance for biodiversity’ through protected areas and OECMs covering at least 30% of terrestrial, inland water, coastal and marine areas by 2030 (CBD, 2022a). The definition of such areas of importance relates very closely to the KBA criteria and specifically references KBAs (CBD, 2024). Hence KBAs are critical in enabling governments to target the designation of new protected areas, the expansion of existing protected areas, or the recognition of OECMs to achieve Target 3, as well as to inform the management actions needed at each site (Plumptre *et al*., 2024a). To this end, it is notable that one‐third (33%) of the 90 countries that had submitted their revised NBSAPs or submitted targets to the Online Reporting Tool of the CBD by March 2025 specifically referenced KBAs, and 23% incorporated KBAs in their national targets. This is now leading to actions to protect and conserve KBAs. For example, the Solomon Islands' NBSAP includes a focus on AZEs which is now leading to the establishment of protected areas (Ministry of Environment, Climate Change, Disaster Management and Meteorology, 2016).

KBAs are also relevant to other targets in the framework. Target 1 requires that all areas are under biodiversity‐inclusive spatial planning and/or effective management processes to bring the loss of ‘areas of high biodiversity importance’ close to zero by 2030. KBAs represent the most comprehensive data set globally for such areas, so incorporating KBAs will strengthen national and sectoral spatial plans. Target 2 aims to restore 30% of degraded terrestrial, inland water, and coastal and marine ecosystems to enhance biodiversity. Restoration of degraded ecosystems in KBAs (and areas connecting them) will help to achieve disproportionate biodiversity benefits. Target 4 calls for urgent management actions to halt human‐induced extinction, reduce extinction risk and achieve recovery of threatened species. Such actions are required within KBAs identified for threatened species and are essential for AZE sites in order to prevent global extinctions given that such sites hold the last remaining populations of one or more Critically Endangered or Endangered species. Target 6 calls for eradicating or controlling invasive alien species ‘especially in priority sites’. KBAs threatened by invasive alien species represent such priority sites (Holmes *et al*., 2019). Hence, protection and effective conservation of KBAs can help to achieve multiple targets simultaneously, reducing costs and enhancing efficiencies. KBAs are also being used to help the private sector measure their risks, dependencies and impacts on biodiversity to achieve Target 15 (see Section XI.6).

### (3) Relevance of KBAs to other international conventions

The World Heritage Convention (WHC) recognises cultural and natural sites of ‘Outstanding Universal Value’. Under WHC criteria, sites may qualify if they ‘contain the most important and significant natural habitats for *in‐situ* conservation of biological diversity, including those containing threatened species of outstanding universal value from the point of view of science or conservation’ or if they are ‘outstanding examples representing significant on‐going ecological and biological processes in the evolution and development of terrestrial, fresh water, coastal and marine ecosystems and communities of plants and animals’ (UNESCO, 2025, p. 30). KBAs are therefore highly relevant in identifying candidate WHC sites and highlighting the specific components of biodiversity of significance within them, as well as for the World Heritage Committee when reviewing proposed natural World Heritage sites. In 76 countries assessed by Foster *et al*. (2010), all 32 of the World Heritage sites based on natural criteria in these countries had been independently identified as KBAs, while an additional six sites identified under geological and aesthetic criteria and 12 sites identified under cultural criteria also qualify as KBAs, suggesting that additional natural criteria could be proposed for these sites.

In the marine realm, KBAs are highly relevant for informing marine spatial planning, protected and conserved area expansion, and management actions. As KBAs are identified as manageable sites, this facilitates direct translation for management actions. Although some marine KBAs are very large (up to 0.7 million km^2^) they are comparable to the 0.3–1.9 million km^2^ extent of the 10 largest marine protected areas. KBAs provided an important input to the CBD‐led process on the description of Ecologically or Biologically Significant Marine Areas (EBSAs), given alignment between the relevant criteria. Some EBSAs were entirely described based on KBAs. Others describe oceanographic processes and are much larger: the largest (11.1 million km^2^) is an order of magnitude larger than the largest KBA. These EBSAs may warrant their own planning processes, which could include protection of the KBAs within them. With the adoption of the Agreement on the Conservation and Sustainable Use of Marine Biological Diversity of Areas beyond National Jurisdiction (the ‘BBNJ Agreement’; United Nations, 2023), there is considerable potential for KBAs to inform the designation of area‐based management tools, including Marine Protected Areas, in areas beyond national jurisdiction.

Freshwater and coastal KBAs are highly relevant for the Ramsar Convention on Wetlands. Some of the criteria for identifying Wetlands of International Importance under the Convention (including those relating to congregations of species) are closely aligned with the KBA criteria, and the importance of KBAs for identifying the gaps and synergies in the network has been recognised by the Convention (Ramsar Convention on Wetlands, 2022).

The relevance of nearly 10,000 KBAs qualifying owing to their importance for migratory species is recognised by the Convention on Migratory Species (CMS; UNEP‐WCMC, 2024) which explicitly references KBAs in several resolutions and technical documents. These include Resolution 12.7 which encourages Parties and other range states to apply the KBA Standard and use ecological networks, such as KBAs, to assess and identify gaps in protected area coverage and secure conservation and sustainable management of these networks. KBAs identified for birds (IBAs) are also important for informing actions under CMS regional flyways agreements, such as the Agreement on the Conservation of African‐Eurasian Migratory Waterbirds (AEWA), which acknowledges that many IBAs serve as essential habitats for migratory waterbirds (AEWA, 2022) and the Memorandum of Understanding on the Conservation of Migratory Birds of Prey in Africa and Eurasia (for which the Annex 3 list of ‘internationally important migratory raptor sites’ is explicitly underpinned by IBAs; Raptors MOU, 2023), as well as the East Asian–Australasian Flyway Partnership (EAAFP), which uses IBAs to support identification of its Flyway Network Sites (EAAFP, 2023). KBA monitoring data facilitate tracking of population trends and assessment of the effectiveness of conservation interventions at a flyway scale, while KBAs are often highlighted for protection and management to support species recovery in action plans under these agreements (e.g. the vulture multi‐species action plan; Botha *et al*., 2017).

### (4) Use of KBAs in indicators tracking progress towards biodiversity commitments

Trends over time in the mean proportion of each KBA covered by protected areas and OECMs is a well‐established indicator tracking progress towards international commitments on biodiversity conservation (Butchart *et al*., 2016). For example, subsets of the indicator have been adopted as official indicators for Sustainable Development Goals 14 and 15 (indicator 14.5.1 for marine, 15.1.2 for terrestrial and freshwater, and 15.4.1 for mountain KBAs), tracking progress towards coverage of important sites for biodiversity by protected areas as a measure of sustainable development (United Nations, 2024). The indicator is also a recommended disaggregation of headline indicator 3.1 (coverage of protected areas) in the monitoring framework of the Kunming–Montreal Global Biodiversity Framework, enabling monitoring of progress towards achieving the component of Target 3 referencing ‘areas of particular importance for biodiversity’ (CBD, 2022b, 2025), while the proportion of KBAs in favourable condition is a complementary indicator for Targets 2 and 3. These indicators extend earlier applications in measuring progress towards CBD targets (Butchart *et al*., 2010, 2019; Secretariat of the Convention on Biological Diversity, 2010, 2020).

Coverage of KBAs by protected areas and OECMs is also used as an indicator by other multilateral environmental agreements. For example, the Convention on Migratory Species (CMS) reported trends over time in protected area coverage of KBAs identified for migratory species in its *State of the World's Migratory Species* report (UNEP‐WCMC, 2024). The UN Convention to Combat Desertification (UNCCD) uses the ‘average proportion of terrestrial KBAs covered by protected areas’ as an area‐based measure of national responses to conserving biodiversity under the convention (UNCCD, 2021). The coverage of wetland KBAs by protected areas is included as indicator in the monitoring framework under development for the 2025–2034 Ramsar Strategic Plan. The coverage of KBAs by protected areas and OECMs has also been reported in a number of international assessments of the state of nature, for example the global and regional assessments of the Intergovernmental Science‐Policy Platform on Biodiversity and Ecosystem Services (e.g. IPBES, 2018, 2019).

### (5) Integration of KBAs into national and sub‐national policies

KBAs are increasingly being reflected in national and sub‐national legislation and policy. Examples come from countries in multiple regions. Greece passed a law in 2023 mandating that all KBAs must be fully covered by protected areas by 2030 (law FEK Α 78/28.03.2023, p. 174, Targets for Conservation of Nature). Two new national marine parks (in the south Aegean sea and Ionian sea) are due to be ratified with a Presidential Decree and will fully cover several KBAs. In Mozambique, the 2022 Ministerial Diploma on Biodiversity Offsets (No. 55/2022) recognises KBAs as avoidance areas under the Environmental Impact Assessment (EIA) process and as priority sites for implementing biodiversity offsets (the first Biodiversity Offset Management Plans in KBAs are already being developed by companies). When EIAs for development projects overlapping KBAs are evaluated by the National Directorate of Environment and Climate Change, they require alternative locations to be considered, or the adequate application of the remaining steps in the mitigation hierarchy and net gain. KBAs have also been formally incorporated into the National Territorial Development Plan (Resolution No. 7/2021), the Marine Spatial Plan (Resolution No. 63/2024), and the Bird Regulation (Decree No. 51/2021) as critical biodiversity zones in which development must be avoided in land and marine use planning and decision‐making. Furthermore, forests located within KBAs are now classified as conservation forests under the new Forest Law (Law No. 17/2023) and its Regulation (Decree No. 78/2024), while all marine and coastal KBAs have been integrated into the National Strategy and Action Plan for marine protected area network expansion, which is pending Ministerial approval. In Uganda, KBAs have been listed as ‘no‐go’ areas for development in their National Biodiversity and Offsets Guidelines (Uganda Ministry of Water and Environment, 2019; NEMA, 2022). In Brazil, the Ministry of Environment and Climate Change declared an ordinance (#287) in 2018 to recognise AZEs as priority sites for conservation and is preparing an ordinance declaring KBAs an official instrument for conservation planning in the country. The government now uses AZE sites when evaluating areas for potential development. For example, in November 2024, a joint declaration by the Ministry of Mines and Energy (MME) and the Ministry of the Environment and Climate Change (MMA) declined to authorise oil and natural gas exploration in a location overlapping three Brazilian AZE sites, including Abrolhos National Marine Park, citing the AZE status to highlight the biodiversity importance of the area (MME and MMA, 2024). The Canadian Government's protected areas strategic plan states explicitly that KBAs will be increasingly used to guide the selection of future National Wildlife Areas (Environment and Climate Change Canada, 2023). In January 2025, the Country Island Complex IBA/KBA was designated as Country Island National Wildlife Area based on its importance for birds as outlined in the IBA/KBA assessment (Government of Canada, 2025). The South African National Biodiversity Institute is integrating KBAs into municipal land‐use planning, the national environmental screening tool, and natural capital accounting (SANBI & UNEP‐WCMC, 2024), with the Environmental Assessment Guideline for Ecosystems formally requiring KBAs to be considered in environmental impact assessments (SANBI, 2023).

### (6) Use of KBAs by the private sector and financial institutions

A recent analysis found that 85% of the world's largest companies that make up the S&P Global 1200 have a significant dependency on nature across their direct operations, with 46% of these companies having at least one asset located in a KBA (S&P Global Sustainable1, 2023). It is therefore unsurprising that private sector organisations are increasingly using KBA data (particularly through the Integrated Biodiversity Assessment Tool; IBAT, https://www.ibat-alliance.org/) when meeting biodiversity standards, frameworks and disclosure requirements, conducting financial and business risk assessments, and informing their strategies and actions relating to biodiversity.

KBAs are referenced in multiple standards and frameworks used by the private sector such as the European Sustainability Reporting Standards under the Corporate Sustainability Reporting Directive, the Taskforce on Nature Related Financial Disclosures, CDP (formerly Carbon Disclosure Project) environmental disclosure system, and Global Reporting Initiative (see online Supporting Information, Table S1). Within these standards and frameworks, business are either required or advised to assess and disclose the impact on KBAs of their direct operations and value chains to inform action that minimises negative biodiversity impacts and maximises positive impacts.

Multiple international financial institutions include KBAs in their safeguarding policies, standards, and investment decisions. For example, the International Finance Corporation's (IFC's) Performance Standard 6 (PS6) and the World Bank's Environment and Social Standard ESS6 include KBAs in their definition of ‘critical habitat’ as areas with high value for the conservation of biodiversity (International Finance Corporation, 2012; World Bank, 2017). If operating in areas of critical habitat, the finance‐receiving organisation has to demonstrate the fulfilment of a range of criteria showing appropriate management of their impact on critical habitat. The IFC regularly seeks advice from the KBA Secretariat when screening projects within or adjacent to KBAs. Several multilateral development banks have policies closely aligned with PS6, and the Equator Principles, a voluntary framework for project finance adopted globally by 131 financial institutions, requires financial institutions to assess, mitigate, and compensate for any potential negative impacts on critical habitat (Equator Principles, 2025). In addition, some multilateral development banks such as the Asian Development Bank (ADB) and the European Bank for Reconstruction and Development (EBRD) have stated in their environmental policies and frameworks that they will not knowingly finance, directly or indirectly, projects that impact AZEs (ADB, 2024; EBRD, 2024).

### (7) Informing donor strategies

KBAs are also widely used by donors to guide where to invest conservation funding (Table S2). For example, the Global Environment Facility (GEF, the financial mechanism for six multilateral conventions) requests applicants seeking funding for the establishment of protected areas to demonstrate that the location qualifies as a KBA. The Critical Ecosystem Partnership Fund targets its investments at priority KBAs within biodiversity hotspots and has strengthened the management and protection of 57 million hectares of KBAs to date. KfW (Kreditanstalt für Wiederaufbau, a German investment and development bank), uses KBAs to help identify ‘Legacy Landscapes’ to which they target funding. In addition, the Bezos Earth Fund, USAID, Alliance Francais pour la Development (AFD), Franklinia Foundation, Hempel Foundation, Forest Foundation Philippines, Foundation for the Philippine Environment, Rainforest Trust, SEE Foundation China, European Union, Global Affairs Canada, Synchronicity Earth and other institutional and individual donors have funded or are funding identification of KBAs (using the global KBA Standard) and their conservation in multiple countries (Table S2).

## XII. EFFECTIVENESS OF KBAS

A small but growing number of studies (mostly focused on birds) sheds light on the effectiveness of KBAs as a means of identifying locations of biodiversity importance and for conserving biodiversity if they are managed appropriately. For example, irreplaceability values (derived from a complementarity‐based analysis) were found to be significantly higher inside than outside KBAs identified for birds in three regions with available data (Australia, southern Africa, and Europe) (Di Marco *et al*., 2015). While most KBAs have been identified for particular species for which they support a significant proportion of the global population, these sites are effective at supporting other species. For example, in the EU, KBAs identified for birds cover 14.5% of land, but a mean of 24.7% of all bird species' ranges and 27.8% of all mammal, amphibian and reptile species' ranges (Kukkala *et al*., 2016), while KBAs worldwide overlap with suitable habitat for 99.7% of bird species (Lansley *et al*., 2025).

Species occurring in KBAs that have higher coverage by protected areas experienced smaller increases in extinction risk over recent decades (measured using the Red List Index): the increase was half as large for bird species with >50% of the KBAs at which they occur completely covered by protected areas, and a third lower for birds, mammals and amphibians restricted to protected AZEs (compared with unprotected or partially protected sites) (Butchart *et al*., 2012, 2016). As a final example, wintering waterbird abundance in Europe and North Africa is higher, and recent trends are more positive, inside IBAs than outside, whether they are legally protected or not (Pavón‐Jordán *et al*., 2020).

## XIII. LIMITATIONS AND CHALLENGES

As with any conservation data set and approach, KBAs have various limitations. The current data set is taxonomically biased, with more sites identified for birds than other groups (although there are more species of plants than birds for which KBAs qualify). There are also relatively few sites identified to date under the criteria relating to ecosystems, ecological integrity and irreplaceability. Many KBA assessments also need updating to reflect the latest information and to determine their global/regional classification, while some assessments lack data on habitats, threats and conservation actions, and some KBAs lack a digitised boundary or have inaccurate boundaries. KBA identification relies on adequate information on potential qualifying species and ecosystems, which may be outdated or insufficient in some cases. KBAs represent a site‐scale approach to conservation, which must be complemented with targeted recovery interventions for many species (Bolam *et al*., 2023) and broader‐scale policy responses addressing land‐use change, agriculture, forestry, fisheries and other drivers of biodiversity loss (Boyd *et al*., 2008). The approach is less useful for species with very large distributions and typically low population densities (because fewer individual sites are globally significant for such species), which applies to many marine species. However, recent work confirms the utility and practicability of KBAs even for broadly distributed and highly mobile marine species such as sharks (Boyd *et al*., 2025). Application of the KBA criteria can be challenging if appropriate data are scarce, while delineating sites as KBAs is more challenging in large areas of broadly homogenous and contiguous habitat.

## XIV. FUTURE PRIORITIES

The KBA initiative has made considerable progress in the decade since the global KBA Standard was published, but much work remains to achieve the KBA Partnership's Vision in which ‘a comprehensive network of sites that contribute significantly to the global persistence of biodiversity is appropriately identified, correctly documented, effectively managed, sufficiently resourced and adequately safeguarded’ (KBA Partnership, 2025b, p. 12). Here, we review priorities for the coming years to 2030.

### (1) Increasing the comprehensiveness of the KBA network

Undertaking comprehensive updates to KBA inventories in those countries with old or incomplete assessments (and in marine areas beyond national jurisdiction) is an urgent priority. This includes reassessing existing KBAs (including for additional taxonomic groups), identifying new KBAs, documenting assessments fully, and applying the criteria relating to threatened or geographically restricted ecosystems, ecological integrity and irreplaceability.

Although KBAs aim to provide a unified currency across the conservation community for important sites for biodiversity, there are also independent taxon‐specific initiatives (e.g. Kor *et al*., 2025; Tetley *et al*., 2022; Kyne *et al*., 2023; IUCN WCPA, 2024). While these can be complementary [e.g. identifying important sites for plant species of socio‐economic significance or highlighting extensive migratory corridors for sharks (Kor *et al*., 2025; Boyd *et al*., 2025)], they typically use expert opinion rather than quantitative thresholds to document significance, and multiple systems and data sets risk creating confusion or complexity for decision‐makers. Further work is needed to explore harmonising these efforts with KBAs as far as possible (Stuart *et al*., 2018, Boyd *et al*., 2025).

### (2) Strengthening tools supporting KBA identification

Further work is needed to enhance the utility of the World Database of KBAs, including developing functionality to support monitoring assessments, management of spatial data on the distribution of KBA‐qualifying species and ecosystems within each site, enhancing reporting functionality to enable users to download data and summary tables, and integration of a ‘scoping tool’ to speed up the identification of species that may meet KBA criteria thresholds at a potential KBA. This latter step is currently time‐consuming because individual sites may be known to support thousands of species, each one of which needs to be assessed for whether the proportion of the global population supported by the site exceeds the relevant KBA criteria thresholds. A KBA scoping tool has been developed to analyse data from the IUCN Red List on the distributions of >90,000 species (those with polygon range maps) and identify which may meet KBA criteria within a site boundary, based on the proportion of their global distribution falling within the area. Its performance was tested by comparing its outputs with outcomes of a manual expert‐led comprehensive assessment of KBAs across seven countries in the Congo Basin and Tropical Andes. The scoping tool successfully identified 93% of 3,145 KBA‐qualifying species that were evaluated by both the tool and experts. Work is underway to make the scoping tool available online for users to access from the World Database of KBAs, and in due course marine species' Area of Habitat maps will be incorporated.

Experience in the application of the global KBA Standard has also indicated that some criteria and/or the guidelines to their application may need to be refined. There is also a need to develop guidelines for identification of KBAs of regional rather than global significance, akin to the guidelines for application of the IUCN Red List criteria at Regional and National Levels (IUCN, 2012).

### (3) Monitoring KBAs

Once KBAs have been identified, it is important to monitor their condition over time to assess whether they are being managed effectively in ways that ensure the persistence of biodiversity for which they have been identified, and if not, to enable actions to be adapted. A standard KBA monitoring protocol has been developed to provide a systematic and straightforward approach for documenting the condition of each site (its state, particularly considering the species and/or ecosystems for which the site qualifies as a KBA), the pressures (threats) acting upon these species and ecosystems, and the conservation actions (responses) being implemented (KBA Partnership, 2025a). KBAs in Kenya have been monitored annually since 2004 using an older version of this protocol, illustrating its practicality and utility for tracking trends and producing policy‐relevant results (Mwangi *et al*., 2010; Gacheru *et al*., 2024), while a similar approach in Australia is used to generate a periodic ‘healthcheck’ of the country's KBAs (Lilleyman, Todd & Maurer, 2024). Once functionality has been developed in the World Database of KBAs to support application of the KBA monitoring protocol, much more extensive monitoring of KBAs should be carried out. Undertaking monitoring assessments requires both automated analysis of remote sensing data (e.g. Beresford, Donald & Buchanan, 2020) and collection of *in‐situ* data (for example to assess levels of hunting or impacts of invasive alien species, and to assess the degree to which management planning is being undertaken and implemented). Such *in‐situ* monitoring is often best undertaken by local communities, including Indigenous Peoples (Crowe *et al*., 2023b). Monitoring data will allow the further development of indicators of the status of KBAs (e.g. the proportion in ‘favourable condition’; CBD, 2022b) that can be used for tracking overall progress in mitigating threats and conserving these sites.

### (4) Effectively conserving and restoring KBAs

Integrating KBAs into countries' NBSAPs and ensuring that these plans are implemented effectively and fully is a critical priority for the KBA initiative. In particular, expanding protected area and OECM networks to cover KBAs and ensuring that their management is effective would help to achieve multiple targets in the Kunming–Montreal Global Biodiversity Framework, as described above. In addition, it is important to restore ecosystems within KBAs where they have become degraded, and to enhance connectivity between KBAs to ensure their continued viability in the long term (Hilty *et al*., 2020). This may require protecting locations between KBAs, restoring degraded habitat, or creating corridors. Integrating KBAs into national sectoral policies (relating to forestry, water, land use, energy, etc.), climate planning (‘nationally determined contributions’ and national adaptation plans) and national development strategies is also critical.

As outlined above, the private sector is increasingly using data on KBAs to assess, disclose and act on the risks from business operations on biodiversity. Strengthening safeguarding policies of financial institutions and extending them to more of these organisations is important because it could have a disproportionately large benefit for conservation of the global KBA network. Similarly, expanding the number of businesses using KBAs to assess, report and minimise their biodiversity impacts will help to avoid damaging developments before they emerge.

### (5) Resourcing KBA data to enable site safeguarding

Achieving these priority actions will have significant financial costs, but these should be seen as effective investments given the wider benefits both to individual actors and society at large. Juffe‐Bignoli *et al*. (2016) estimated that the annual maintenance costs for the KBA data set were USD 0.86 M in 2013, with a further USD 0.6 M invested annually in volunteer contributions. In 2025, the annual budget of the KBA secretariat totalled USD 1.2 M, including the costs of Regional Focal Points to support NCGs and individuals undertaking KBA assessments, staff costs for reviewing and confirming sites proposed as KBAs, providing training, communications, maintaining and developing the World Database of KBAs and the KBA website, data management, and policy support. In addition, the KBA Partnership invests USD 3–5 M annually in activities to deliver the KBA strategy, including support to the KBA secretariat and database, training, support to KBA NCGs, and engagement with governments and the private sector (KBA Partnership, 2024b). Juffe‐Bignoli *et al*. (2016) also estimated that over USD 100 M has been invested since 1979 in developing the KBA data set, with most of this (66%) provided by philanthropic sources, followed by government (27%). They estimated that it would cost USD 21 M to complete comprehensive assessments of KBAs worldwide (and annual costs would increase to USD 2 M per year once this had been achieved). These are arguably modest sums relative to the benefits provided to a wide range of stakeholders, from governments to businesses.

Effectively conserving the global network of KBAs will require substantially greater resources. McCarthy *et al*. (2012) estimated that protecting and effectively managing a comprehensive global network of terrestrial KBAs would cost USD 76.1 billion annually (of which USD 22.4 billion annually would be for the costs in lower income countries). However, these costs are dwarfed by the value of the potential goods and services that biodiversity provides (including climate change mitigation), hence they can be seen as highly cost‐effective investments (McCarthy *et al*., 2012).

## XV. CONCLUSIONS

(1) Nearly a decade on from the publication of the global KBA Standard and launch of the KBA Partnership, much has been achieved in terms of developing the tools, processes, systems and structures to support KBA identification and mainstream KBAs into policy processes across government and businesses.
(2) A growing number of countries have completed relatively comprehensive KBA assessments, while many more have embarked on this exercise. Simultaneously, KBAs are being increasingly recognised in multiple policy processes, and are influencing decisions from designation of protected areas to lending of development finance.
(3) KBAs represent one tool in the conservation toolbox, albeit a particularly useful and multifunctional one, alongside landscape‐scale restoration, targeted species recovery actions, threat mitigation and other approaches.
(4) The urgency to identify the remaining sites of greatest importance for biodiversity and protect, conserve and safeguard them effectively has never been greater.

## Supporting information


**Table S1.** Relevance of Key Biodiversity Areas (KBAs) to the private sector.
**Table S2**. Donors supporting Key Biodiversity Area (KBA) identification and conservation.

## Data Availability

The data that support the findings of this study are available from https://www.keybiodiversityareas.org or are available from the corresponding author upon reasonable request.
