# Supplementary material for: Extent, characteristics and policy applications of Key Biodiversity Areas
Source: Biol Rev Camb Philos Soc. 2026 Feb 19;101(4):1735–59. doi: 10.1002/brv.70144 (PMC13326769; doi:10.1002/brv.70144)
Supplement: Supplementary file 1 — Table S1. Relevance of Key Biodiversity Areas (KBAs) to the private sector. Table S2. Donors supporting Key Biodiversity Area (KBA) identification and conservation. [file BRV-101-1735-s001.docx]

**SUPPORTING INFORMATION**

**Table S1.** Relevance of Key Biodiversity Areas (KBAs) to the private sector.

| **Standard/framework/ institution** | **Notes** |
| --- | --- |
| Asian Development Bank (ADB) | The most recent draft of the ADB environmental and social standard 6 identifies KBAs as priority features and uses many of the KBA criteria to define Critical Habitat and the mitigation actions needed in such habitat. ADB environmental policies and frameworks state that ADB will not knowingly finance, directly or indirectly, projects that impact AZEs. |
| European Sustainability Reporting Standards (ESRS) under the Corporate Sustainability Reporting Directive | ESRS E4 requires companies to disclose “sites in its own operations that are in or near biodiversity sensitive areas” and “the activities negatively affecting those biodiversity-sensitive areas”. KBAs are included in the definition of “biodiversity sensitive areas”. |
| CDP (formerly Carbon Disclosure Project) | KBAs are referenced in CDP’s corporate questionnaire as a “type of area important for biodiversity”. The questionnaire requires companies to assess and disclose whether any of their activities are located in or near KBAs, and if so, to provide additional information on proximity and mitigation measures. |
| European Bank for Reconstruction and Development (EBRD) | EBRD environmental policies and frameworks state that they will not knowingly finance, directly or indirectly, projects that impact AZEs. |
| EU Taxonomy | The EU taxonomy is a classification system that defines criteria for economic activities that are aligned with a net zero trajectory by 2050. It helps direct investments to the economic activities most needed for the transition, in line with the European Green Deal objectives. The taxonomy forms a key part of the EU’s sustainable finance framework. It contains over 80 references to KBAs, requiring mitigation measures to be implemented to safeguard these sites from harmful impacts of developments (EU Technical Expert Group on Sustainable Finance, 2020). |
| Equator Principles | The Equator Principles is a voluntary framework for project finance adopted globally by 131 financial institutions. The principles require financial institutions to assess, mitigate, and compensate for any potential negative impacts on critical habitat. KBAs are identified as likely Critical Habitat. |
| Global Reporting Initiative (GRI) | The GRI 101: Biodiversity 2024 standard identifies KBAs as a type of “ecologically sensitive area”. To align with GRI 101, organisations must assess which of their sites are in or near ecologically sensitive areas. such as KBAs. For any sites near a KBA, additional information on proximity is required. |
| International Finance Corporation Performance Standard 6 | KBAs are included in the definition of “critical habitat” in PS6 as areas with high value for the conservation of biodiversity. Operators in critical habitat must demonstrate the fulfilment of a range of criteria showing appropriate management of their impact on critical habitat. |
| Taskforce on Nature Related Financial Disclosures (TNFD) | The TNFD identifies KBAs as a type of “ecologically sensitive location”. Companies should assess which of their sites are in or near ecologically sensitive locations and prioritise these for further data collection and action. |
| World Bank Environment and Social Standard ESS6 | The World Bank requires borrowers to ensure that any activities undertaken in a KBA are consistent with its legal protection status and management objectives. Borrowers must also identify and assess potential project-related adverse impacts and apply the mitigation hierarchy so as to prevent or mitigate adverse impacts from projects that could compromise the integrity, conservation objectives or biodiversity importance of a KBA. |

**Table S2.** Donors supporting Key Biodiversity Area (KBA) identification and conservation.

| **Donor** | **Notes** |
| --- | --- |
| Alliance Francais pour la Development (AFD) (https://www.afd.fr/en) | AFD is supporting updates to the KBA networks in South Africa, Namibia, Malawi and Mozambique. |
| Bezos Earth Fund (https://www.bezosearthfund.org) | Bezos Earth Fund supported the updating of KBA networks in three countries in the Congo Basin and four in the Andes region, including development of the World Database of KBAs. |
| Critical Ecosystem Partnership Fund (CEPF) (https://www.cepf.net) | CEPF targets its investments at priority KBAs within biodiversity hotspots and has strengthened the management and protection of 57 million hectares of KBAs to date. |
| European Union (EU) (https://environment.ec.europa.eu/funding_en) | As well as supporting KBAs through the CEPF, the EU considers KBAs as important sites for action in their “Larger than tigers”, “Larger than elephants” and “Larger than jaguars” strategies for Asia, Africa and Latin America respectively. |
| Forest Foundation Philippines (https://www.forestfoundation.ph) | Forest Foundation is funding updates to the terrestrial and marine KBA network in the Philippines. |
| Foundation for the Philippine Environment (https://www.fpe.ph) | FPE are funding updates to the terrestrial and marine KBA network in the Philippines. |
| Franklinia Foundation (https://fondationfranklinia.org/en) | Franklinia Foundation uses the number of KBAs established for trees as one of its indicators of impact. |
| Garfield Weston Foundation (GWF) (https://garfieldweston.org) | GWF have supported the development of the World Database of KBAs. |
| Global Affairs Canada (https://international.canada.ca/en/global-affairs) | Global Affairs Canada supported updates to the KBA network in Canada. |
| Global Environment Facility (GEF) (https://www.thegef.org) | GEF includes several multilateral funds to address the planet’s most pressing environmental challenges. It requests applicants seeking funding for the establishment of protected areas to justify that the location qualifies as a KBA. |
| Hempel Foundation (https://hempelfoundation.com) | Hempel Foundation is supporting updates to the KBA network in Tanzania and uses KBAs to guide its investments in biodiversity conservation. |
| Kreditanstalt für Wiederaufbau (Kfw) (https://www.kfw.de/kfw.de.html) | KfW is a German investment and development bank that uses KBAs to help identify “Legacy Landscapes” to which they target funding. |
| Marks Family Charitable Trust (https://www.marksfamilycharitabletrust.com) | Marks Family Charitable Trust has supported the development of the World Database of KBAs. |
| Rainforest Trust (https://www.rainforesttrust.org) | Rainforest Trust supports the creation of new protected areas or other effective area-based conservation measures (OECMs) and uses KBAs to help target where it invests. |
| SEE Foundation China (http://foundation.en.see.org.cn/about/foundation) | SEE Foundation is supporting training and updates to China’s KBA network. |
| Synchronicity Earth ([https://www.synchronicityearth.org/](https://url.uk.m.mimecastprotect.com/s/CRSwC1Pou6kmOMiGhACV2kHL?domain=synchronicityearth.org/)) | Synchronicity Earth supported updates to the global network of freshwater KBAs. |
| The Nature Conservancy (TNC) ([https://www.nature.org/en-us/](https://url.uk.m.mimecastprotect.com/s/9WsYCZNKh7DRx5SzflCB47lM?domain=nature.org/)) | TNC supported KBA identification in Lake Tanganyika (Burundi, Tanzania, Zambia and DR Congo). |
| USAID (https://www.usaid.gov) | USAID was supporting KBA network updates in several countries including Mozambique and Uganda before the agency’s operations ceased. |

**References**

EU Technical Expert Group on Sustainable Finance (2020) Taxonomy report: technical annex. Updated methodology & Updated Technical Screening Criteria. Available at: <https://finance.ec.europa.eu/system/files/2020-03/200309-sustainable-finance-teg-final-report-taxonomy-annexes_en.pdf>.
